# Carbenaporphyrins: No Longer Missing Ligands in N‐Heterocyclic Carbene Chemistry

**DOI:** 10.1002/anie.202013434

**Published:** 2020-11-27

**Authors:** Theo Maulbetsch, Doris Kunz

**Affiliations:** ^1^ Institut für Anorganische Chemie Eberhard Karls Universität Tübingen Auf der Morgenstelle 18 72076 Tübingen Germany

**Keywords:** carbazole, lithium, N-heterocyclic carbene, porphyrin, scandium

## Abstract

The synthesis of an NHC‐containing porphyrinoid ligand is presented. The formally antiaromatic 20 πe^−^ macrocyclic framework can be obtained via a 1,3‐dipolar cycloaddition (“click‐reaction”) to form two triazole moieties which were alkylated to the respective triazolium macrocycle. Deprotonation of the ligand precursor with lithium bases to the respective dilithio carbenaporphyrin complex and transmetallation to scandium lead to complexes that exhibit orange fluorescence. Optical property combined with TD‐DFT studies verify an aromatic character for each heterocyclic moiety rather than an antiaromatic macrocycle in the ligand precursor as well as in the complexes. While the geometric features of the carbenaporphyrin ligand strongly resemble those of porphyrin, DFT calculations reveal a stronger electron‐donating ability of the new ligand.

## Graphical abstract

The synthesis of a carbenaporphyrin ligand based on carbazole and triazolylidene is reported. Instead of a macrocyclic aromatic or antiaromatic character, the aromaticity of each heterocyclic moiety is preserved, which results in optical properties different from porphyrins, for example, fluorescence. In Li^+^ and Sc^3+^ complexes the ligand reveals striking geometric similarity to porphyrins, but is a stronger electron donor.

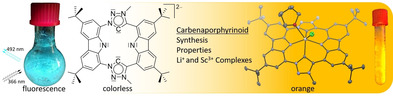

Porphyrins are an abundant ligand class in nature as well as within coordination chemistry.^[1]^ Complexes of almost every metal ion are known with these ligands, whose diverse properties also define the reactivity and application, for example, catalysis,^[2]^ supramolecular chemistry,^[3]^ chemo sensors,^[4]^ and organic electronics.^[5]^ The aromatic 18 πe^−^ macrocyclic structure (Figure 1) has been adapted by nature as well as by chemists varying the substituents at the porphyrin ring,^[6]^ synthesizing expanded and contracted variations[^7^, ^8^] as well as altering the nature of the donor atoms or the position of the heteroatoms.^[8]^ An interesting example of the latter are so‐called N‐confused porphyrins,^[9]^ in which one or two C atoms instead of the N atoms of the pyrrole moieties are coordinated to the metal. Thus, the carbon donor atom gains carbene character.^[10]^

**Figure 1:**
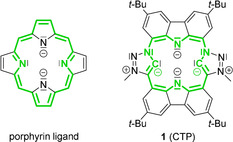
The regular porphyrin ligand (left) and our carbazole‐triazolylidene porphyrin (CTP) **1**. The conjugated π‐system (green) is Hückel‐aromatic in porphyrin (18 e^−^), and formally antiaromatic in **1** (20 e^−^).

N‐heterocyclic carbene ligands (NHCs) are stable (“bottleable”) singlet carbenes^[11]^ that show strong overall donor‐properties which can be beneficial in coordination chemistry[^12^, ^13^] and catalysis.^[14]^ They are suitable to build up π‐conjugated poly(NHC) ligands and porphyrin‐fused NHCs.^[15]^ A porphyrin with an embedded NHC moiety is also known.^[16]^ The idea to not only invert pyrrole moieties of the porphyrin but to substitute them by NHCs has existed for a long time^[17]^—however, its realization has remained elusive. Our own early research on this topic showed that precursors based on methylene‐connected pyrrole and imidazolium units are not suitable for this purpose owing to an elimination reaction upon deprotonation[^18^, ^19^] and therefore, we introduced a carbazole moiety^[20]^ in which connecting sp^2^ carbon atoms prevent this elimination.^[18]^ This still led to major synthetic problems in constructing an alternating imidazolium–pyrrole macrocycle.^[21]^ However, triazole‐ instead of imidazole‐based NHCs are readily accessible by a 1,3‐dipolar cycloaddition[^22^, ^23^, ^24^] and thus could be used to build up the desired macrocycle **1** based on carbazole **2** using compounds **3** and **4** as building blocks (Scheme 1). After initial attempts^[25]^ we set out again to synthesize the formally antiaromatic (20 πe^−^) carbazole‐triazolylidene porphyrin (CTP) **1**. The synthesis of **1** and the investigation of its special properties are the objectives of this work.

**Scheme 1:**
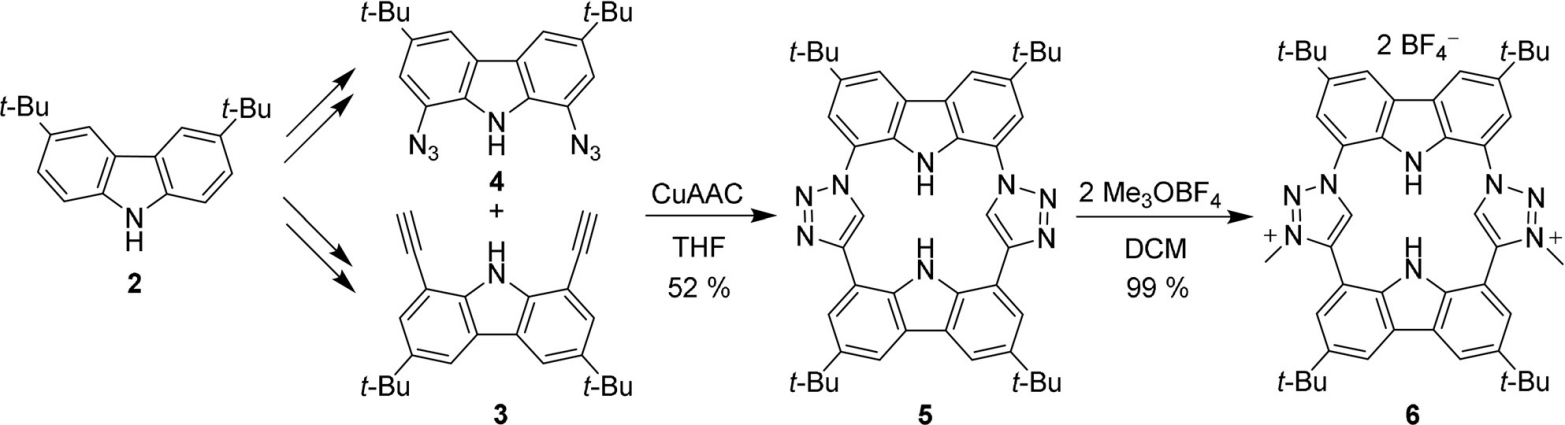
Synthesis of macrocycle **5** in a CuAAC reaction of **3** and **4**, which are both derived from carbazole **2**, and methylation to the CTP precursor **6**.

The key step of the synthesis is the copper‐catalyzed alkyne–azide cycloaddition (CuAAC),^[26]^ the so‐called click‐reaction,^[27]^ in which both triazole moieties are built up under formation of the macrocycle. Both alkyne **3** and azide **4** are literature‐known compounds and can be obtained from carbazole **2**.[^28^, ^29^] The azide formation from the respective 1,8‐bromocarbazole did not work in our hands following the literature procedure.^[29]^ Instead, we applied a Sandmeyer‐type reaction^[30]^ and succeeded in isolating **4** in 93 % yield from the respective 1,8‐diaminocarbazole as a kinetically stable (slow decomposition above 100 °C) but light sensitive product. The *tert*‐butyl groups in **2** are not only beneficial as protecting groups but also enhance the solubility of the product.

The copper‐catalyzed 1,3‐dipolar cycloaddition to form the triazole macrocycle **5** was described by Arnold to proceed only with 17 % yield using a high catalyst loading and TBTA (tris((1‐benzyl‐1*H*‐1,2,3‐triazol‐4‐yl)methyl)amine) as ligand.^[31]^ After optimizing the conditions of this reaction as regards concentration, solvent, and stoichiometry of the reactants, we were able to isolate macrocycle **5** in 52 % yield as a colorless crystalline product.

The ^1^H NMR spectrum ([D_8_]THF) shows five aromatic signals of equal ratio (four from the chemically inequivalent carbazoles at 8.10, 8.18, 8.24, and 8.41 ppm, and one from the two equivalent triazole moieties at 9.82 ppm), two broad N−H signals (10.19 (Carb‐N) and 9.43 ppm (Carb‐C)) and two singlets for the *tert*‐butyl groups (1.54 and 1.56 ppm). X‐ray structure analysis of a colorless single crystal—obtained from slow evaporation of a solution in tetrahydrofuran—confirms the identity of **5** (Figure 2).^[32]^ The absence of color already indicates that an aromatic 18 e^−^ annulene core, like in porphyrin, cannot be expected. Indeed, the individual aromaticity of the carbazole and triazole moieties is retained, which results, in addition to steric congestion of the C−H and N−H protons, in a small inclination of the carbazole planes (9.2°) and a larger of the triazole planes (72.3°) into the opposite direction (tilting of the triazole plane against the carbazole plane 36° (mean)), so that the C−H and N−H groups are pointing away from each other. It is enhanced by N−H hydrogen bonds to one molecule of tetrahydrofuran. This coordination provides an explanation for the broadened N−H signals in the ^1^H NMR spectrum.

**Figure 2:**
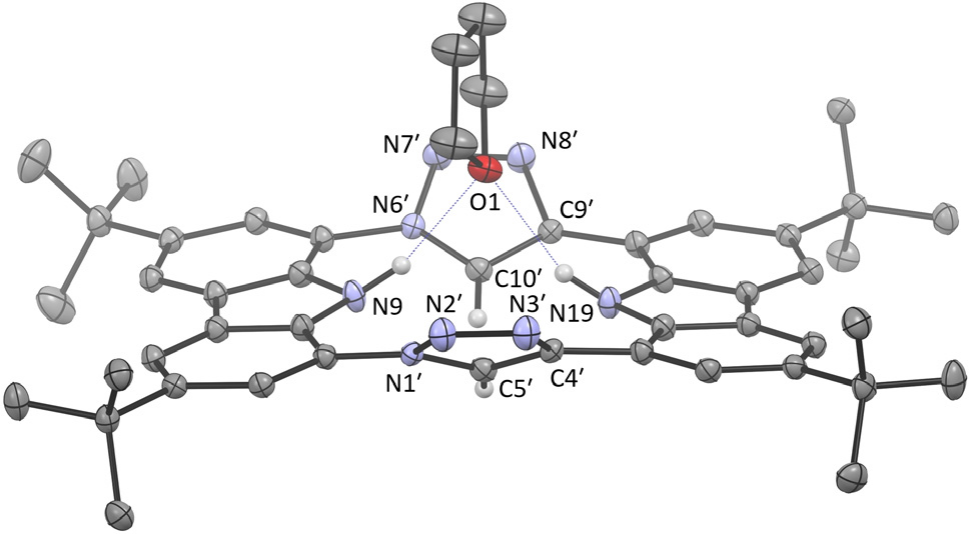
The solid‐state molecular structure of macrocycle **5** (anisotropic atomic displacement parameters at 50 % probability level). Hydrogen atoms (except for the N−H and triazole C−H) and three co‐crystallized THF molecules are omitted for clarity. One THF is hydrogen‐bonded (O1–H9=2.095 Å, O1–H19=2.171 Å).

The alkylation of **5** to form the triazolium moieties in **6** did not proceed with methyl iodide but required the stronger Meerwein's salt (trimethyloxonium tetrafluoridoborate). The dicationic macrocycle **6** was obtained quantitatively as a colorless crystalline material. In the ^1^H NMR spectrum product **6** is identified by the signal of the methyl groups at 4.55 ppm. Although the N−H groups are chemically inequivalent, only one (broad) signal (2 H) at 9.40 ppm is observed, possibly due to fast exchange. The downfield‐shifted signal of the triazolium C−H moiety at 9.92 ppm, which indicates a higher acidic character of the hydrogen atom than in **5**, and its carbon chemical shift at δ(^13^C)=132.2 ppm compared to 124.8 ppm in **5** are very characteristic features.

The X‐ray structure analysis of single crystals obtained from slow evaporation of a solution in tetrahydrofuran confirms the successful formation of the triazolium macrocycle **6**.^[32]^

Similar to macrocycle **5**, hydrogen bonding to the acidic hydrogen atoms is observed, which in the case of **6** also includes the triazolium hydrogen atoms. They have short contacts to the tetrahydrofuran oxygen, while the carbazole N−H atoms are hydrogen‐bonded to the fluorine atom of one of the BF_4_^−^ counterions (Figure 3). As already recognized in **5**, the carbazole planes are only slightly tilted toward each other, while the hydrogen bonding to the fluorine atom is realized through a small tetrahedralization of the nitrogen atoms (sum of angles at N9 and N19=350°). The triazolium planes are inclined by 87.1° and are tilted by an average of 44.1° against the carbazole planes (mean values).

**Figure 3:**
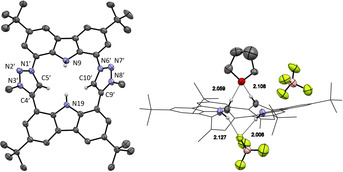
Two views of the solid‐state molecular structure of the triazolium macrocycle **6** (anisotropic atomic displacement parameters at 50 % probability level). Left: hydrogen atoms (except for the acidic N−H and triazolium C−H), the two BF_4_^−^ counterions and one hydrogen bonded THF molecule are omitted for clarity. Right: Hydrogen bonding between one BF_4_^−^ counterion to the carbazole N−H and between one THF and the triazolium hydrogen atoms. The macrocycle is depicted in wireframe for clarity reasons.

Like macrocycle **5** the CTP precursor **6** is a colorless compound, (porphin is dark red), which indicates that **6** lacks antiaromatic character (20 e^−^; for example, isophlorin^[33]^) nor is it a macrocyclic aromatic π‐system like porphyrin (18 e^−^). Instead, it resembles other carbazole porphyrinoids regarding this feature.[^34^, ^35^, ^36^, ^37^, ^38^, ^39^, ^40^, ^41^] In the UV/Vis spectrum (THF) an absorption maximum at 359 nm is detected, while it lacks the characteristic Soret band at 400–450 nm of porphyrins that originates from a π–π* transition of the delocalized 18 e^−^ aromatic ring system.^[42]^ DFT calculations confirm that the carbazole and NHC moieties keep their individual aromatic character and only very small contributions to the molecular orbitals of the other moieties are observed, for example, the HOMO is almost fully localized on one carbazole moiety (Figure 4). TD‐DFT calculations indicate that the first relevant absorption maximum at 359 nm consists of electronic transitions from a carbazole‐centered orbital (HOMO−3) to an unoccupied orbital of the triazole moieties (LUMO+1).

**Figure 4:**
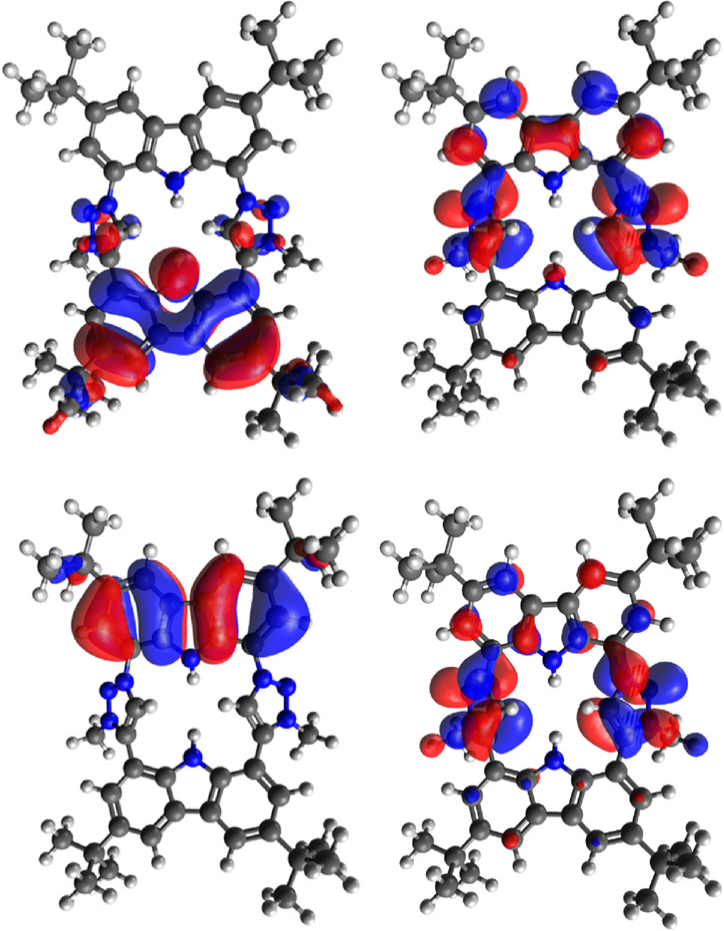
HOMO (top left) and LUMO (top right) of **6** and the orbitals HOMO−3 (bottom left) and LUMO+1 (bottom right) whose transition (374 nm) contributes strongest to the absorption at 359 nm (isosurface 0.02).

It is comprehensible that **6** exhibits fluorescence. Excitation at 358 nm in THF leads to a broad emission band with a maximum at 553 nm. In the solid state the excitation band is broader, and the maximum shifted to 380 nm. Irradiation at 380 nm leads to a hypsochromic shift of the emission maximum to 492 nm (Figure 5).

**Figure 5:**
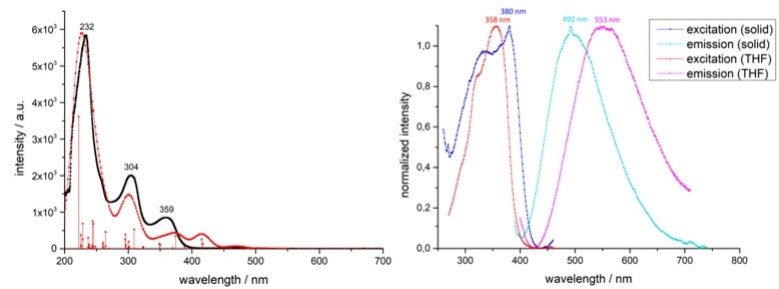
Left: experimental (black) and calculated (red) absorption spectrum of **6** (TD‐DFT B3LYP DEF2‐TZVP, CPCM(THF)). Right: excitation and emission spectra of **6** in solid state (excitation at 380 nm) and in solution (excitation at 358 nm; tetrahydrofuran, *c*=5×10^−5^ M).

As the carbazole N−H moieties are more acidic than the triazolium C−H moieties, formation of the mesoionic carbene cannot be achieved without deprotonation of the carbazole, so that a free carbene carbazole porphyinoid “**1⋅2 H^+^**” is not accessible. To elucidate the properties of the “free” dianionic CTP ligand **1**, we calculated its structure and the photochemical properties by means of DFT (Supporting Information). The classic MOs of carbene σ‐character are lower in energy (HOMO−2 and HOMO−3) than two occupied π‐orbitals of the carbazole moieties (Supporting Information). The calculated UV spectrum shows a relevant absorption at 435 nm, which would result in an orange color of the “free” CTP ligand.

While other carbazole porphyrinoids can be oxidized experimentally to aromatic 18 πe^−^ species,[^34^, ^35^, ^36^, ^37^, ^38^, ^39^, ^40^, ^41^] already calculations of the oxidized CPT ligand **1** with a macrocyclic 18 e^−^ π‐system did not converge to a stable minimum structure nor did the oxidation product of **6** lead to a singlet ground state (Supporting Information).

Deprotonation of macrocycle **6** with 4 equiv of lithium bis(trimethylsilyl)amide led to isolation of the carbene porphyrine dilithium complex **7** as a yellow solid along with two equivalents of LiBF_4_, which was not removed due to its similar polarity (Scheme 2). Complex **7** was identified in the ^1^H NMR spectrum via the absence of the N−H and triazole C−H peaks as well as a high‐field shift of the aromatic signals of the two carbazole moieties (7.69, 8.03, 8.22 (2×) ppm). In the ^13^C NMR spectrum, the signal of the mesoionic carbene carbon atom is detected at 187.9 ppm, which is 13–18 ppm lower than that of typical free mesoionic carbenes (201–206 ppm).[^22^, ^23^] This fits well with the expected incremental chemical shift upon coordination of lithium.[^12^, ^43^] In the ^7^Li NMR spectrum, a broad peak at 0.6 ppm indicates a fast ion exchange between complex **7** and LiBF_4_. Cooling to −80 °C slows down this exchange so that separate signals for complex **7** at typical 1.90 ppm^[44]^ and LiBF_4_ (−0.6 ppm) can be observed. According to DFT calculations each lithium atom is coordinated in an N,C,N‐η^3^‐coordination mode by the CTP ligand and by two THF molecules, resulting in a *C*_2_ symmetric complex (see Supporting Information). In the known dilithio tetraphenylporphyrin, an η^4^‐coordination of the pyrrole nitrogen atoms to both lithium ions as well as coordination of one Et_2_O molecule per lithium atom was revealed by X‐ray structure analysis.^[45]^ In the ^13^C NMR spectrum of complex **7** at −80 °C the simple signal set of the *C*_2_ symmetric complex is observed. The carbene signal is broadened but the ^1^ *J*_LiC_ coupling is not resolved (which is typical for Li carbene complexes due to fast Li exchange).

**Scheme 2:**
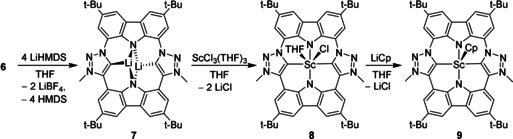
Deprotonation of the macrocycle **6** to yield the dilithium complex **7** and synthesis of the CTP scandium complexes **8** and **9**. Likely, two THF molecules are coordinated to each of the lithium atoms in complex **7**.

Transmetallation of **7** with scandium trichloride in tetrahydrofuran resulted in an orange solution of the desired scandium porphyrin complex **8** (Scheme 2) that exhibits orange fluorescence under UV light (366 nm). Compared to the dilithium complex **7**, the signals of the aromatic protons in the ^1^H NMR spectrum are slightly shifted to lower field, which coincides well with the increased Lewis acidity of scandium. Due to the strong quadrupole moment of the scandium nucleus (^45^Sc: *I*=7/2), the carbene signal could not be detected. However, a ^45^Sc NMR spectrum confirms the formation of a new scandium complex with a signal at 285.0 ppm, while the signal of the starting material [ScCl_3_(THF)_3_] (217.8 ppm) is no longer observed.

All attempts to isolate complex **8** by removal of the solvent resulted in decomposition of this highly moisture sensitive complex. However, from a supersaturated solution of **8** in tetrahydrofuran, orange single crystals were obtained and subjected to X‐ray structure analysis.^[32]^ The molecular structure (Figure 6) confirms the η^4^‐coordination mode of the CTP ligand to scandium by substitution of two chlorido and two tetrahydrofuran ligands. The CTP ligand takes in a basal coordination, so that the scandium is located 0.99 Å above the plane spanned by the coordinating atoms N9‐C5′‐N19‐C10′. This coordination is typically found in porphyrin lanthanoid complexes, which leads to *cis* coordination of additional ligands.^[46]^ Only two porphyrin scandium chlorido complexes (intense purple) have been structurally characterized so far.[^47^, ^48^] Both have a coordination number of 5 and the Sc is located 0.6–0.7 Å above the porphyrin coordination plane. In complex **8** the additional tetrahydrofuran ligand leads to a coordination number of 6 in a distorted prismatic arrangement. The carbazole planes are almost coplanar (4.0°) and the inclination of the triazole planes is reduced to only 44.1°. They are tilted against the carbazole planes by 22° (mean). The angles at the carbene atoms (N1′‐C5′‐C4′ 102.8°, N6′‐C10′‐C9′ 103.4°) are reduced (by 3°) compared to the carbene precursor **6** as it is typically found for NHC ligands and their respective azolium precursors.

**Figure 6:**
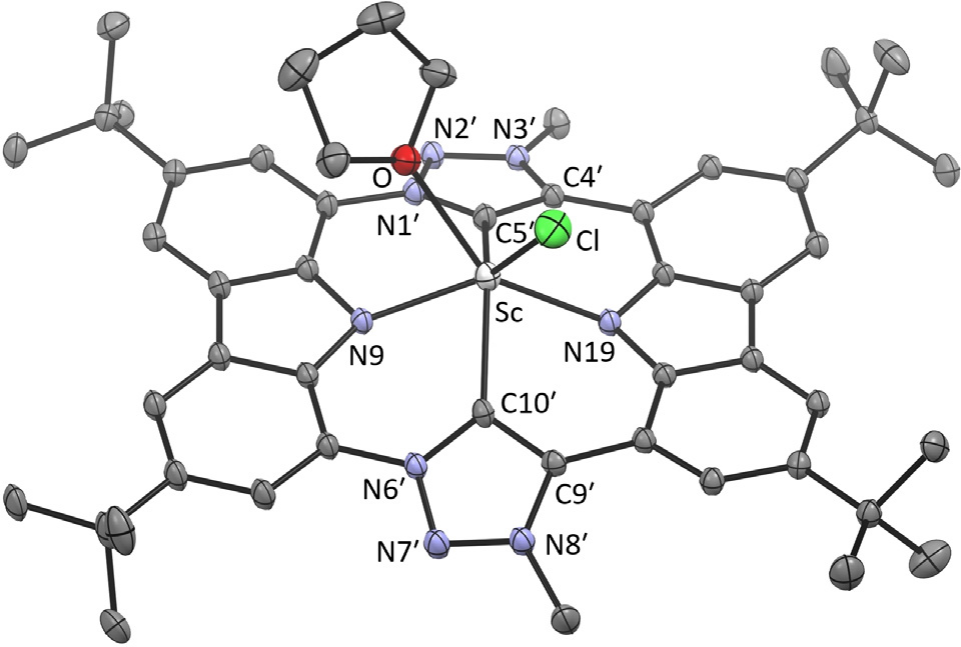
The solid‐state molecular structure of **8**. Anisotropic atomic displacement parameters shown at 50 % probability and hydrogen atoms are omitted for clarity.

While the geometric features of the CTP coordination to Sc are similar to that of the porphyrin ligand, the electronic properties of the carbene moiety should impart a stronger electron‐donating character to the CTP ligand. Therefore, we calculated the Mulliken charge of complex **8** and the porphyrin Sc complex TTPScCl^[48]^ with and without coordinated THF. In both cases, the scandium atom is significantly less positively charged in the CTP than in the porphyrin complexes (Figure 7).

**Figure 7:**
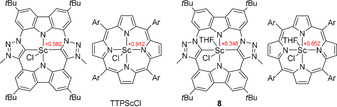
Comparison of the Mulliken population analysis of the scandium metal centers between **8** and TTPScCl (Ar=*p*‐tolyl). The optimized structures (BP86/def2‐TZVP) are identical within the error of the X‐ray structures available.

It is known from the mixed Cp‐porphyrin sandwich complex [ScCp(OEP)] (OEP=octaethylporphyrin)^[49]^ that the macrocyclic aromatic ring current exerts a strong shielding effect on the Cp protons (*δ*(^1^H)=1.68 ppm). To probe a potential ring current in our CTP ligand, we added CpLi to a solution of complex **8** in [D_8_]THF (Scheme 2). The formation of complex **9** is detected in the ^45^Sc NMR with a signal at 136.4 ppm, which is about 150 ppm at higher field than that of **8** and a good indicator for the substitution by a Cp ring.^[50]^ The Cp−H signal is detected at *δ*(^1^H)=5.21 ppm (LiCp: *δ*(^1^H)=5.69 ppm), which is comparable to other CpSc complexes.^[51]^ This clearly precludes any macrocyclic aromatic or antiaromatic ring current effect. Complex **9** can also be synthesized in a one‐pot procedure starting from the ligand precursor **6** in 80 % isolated yield as an orange solid. The color results from a weak absorption with a maximum at 485 nm and a stronger one with a maximum at 408 nm in THF. The complex shows orange fluorescence in the solid state (*λ*_exc_=449 nm, *λ*_em_=614 nm) and yellow fluorescence in solution (*λ*_exc_=411 nm, *λ*_em_=517 nm (THF)) (Supporting Information).

To conclude, we have synthesized and investigated the properties of the carbenaporphyrin ligand **1** (CTP) and its lithium and scandium complexes. A potential aromatic or antiaromatic character of an 18 or 20 πe^−^ macrocycle in the carbene precursor **6** or in the complexes **7**–**9** can be excluded. Instead, the heterocyclic moieties keep their individual aromaticity, but provide the geometric features of porphyrins upon complexation with lithium and scandium. Thus, a porphyrinoid character can be attributed to ligand **1**, and in addition it features stronger electron‐donor properties than porphyrins owing to its mesoionic NHC moieties. The influence of the electronic properties on the reactivity of these complexes and on those with redox‐active metal centers is subject of our current research.

## Conflict of interest

The authors declare no conflict of interest.

## Supporting information

As a service to our authors and readers, this journal provides supporting information supplied by the authors. Such materials are peer reviewed and may be re‐organized for online delivery, but are not copy‐edited or typeset. Technical support issues arising from supporting information (other than missing files) should be addressed to the authors.

Supplementary

## References

[1] A. R. Battersby , Nat. Prod. Rep. 2000, 17, 507. 11152419 10.1039/b002635m

[2a] B. Meunier , Chem. Rev. 1992, 92, 1411;

[2b] C.-M. Che , J.-S. Huang , Chem. Commun. 2009, 3996; 10.1039/b901221d 19568617

[2c] T. Chatterjee , V. S. Shetti , R. Sharma , M. Ravikanth , Chem. Rev. 2017, 117, 3254. 27813402 10.1021/acs.chemrev.6b00496

[3] S. Liu , D. V. Kondratuk , S. A. L. Rousseaux , G. Gil-Ramírez , M. C. O'Sullivan , J. Cremers , T. D. W. Claridge , H. L. Anderson , Angew. Chem. Int. Ed. 2015, 54, 5355; 10.1002/anie.201412293 PMC4471551 25683453

[4] Y. Ding , W.-H. Zhu , Y. Xie , Chem. Rev. 2017, 117, 2203. 27078087 10.1021/acs.chemrev.6b00021

[5a] G. Calogero , A. Bartolotta , G. Di Marco , A. Di Carlo , F. Bonaccorso , Chem. Soc. Rev. 2015, 44, 3244; 25855097 10.1039/c4cs00309h

[5b] A. Kay , M. Graetzel , J. Phys. Chem. 1993, 97, 6272.

[6a] C. M. B. Carvalho , T. J. Brocksom , K. T. de Oliveira , Chem. Soc. Rev. 2013, 42, 3302; 23364186 10.1039/c3cs35500d

[6b] S. Hiroto , Y. Miyake , H. Shinokubo , Chem. Rev. 2017, 117, 2910. 27709907 10.1021/acs.chemrev.6b00427

[7a] J. Mack , Chem. Rev. 2017, 117, 3444; 28222605 10.1021/acs.chemrev.6b00568

[7b] T. Tanaka , A. Osuka , Chem. Rev. 2017, 117, 2584; 27537063 10.1021/acs.chemrev.6b00371

[7c] T. Sarma , P. K. Panda , Chem. Rev. 2017, 117, 2785. 28005351 10.1021/acs.chemrev.6b00411

[8] T. D. Lash , Synlett 2000, 2000, 279.

[9a] H. Furuta , T. Asano , T. Ogawa , J. Am. Chem. Soc. 1994, 116, 767;

[9b] P. J. Chmielewski , L. Latos-Grażyński , K. Rachlewicz , T. Glowiak , Angew. Chem. Int. Ed. Engl. 1994, 33, 779;

[9c] H. Maeda , A. Osuka , H. Furuta , J. Am. Chem. Soc. 2003, 125, 15690. 14677928 10.1021/ja038519t

[10] A. Ghosh , Angew. Chem. Int. Ed. Engl. 1995, 34, 1028;

[11a] A. J. Arduengo , R. L. Harlow , M. Kline , J. Am. Chem. Soc. 1991, 113, 361;

[11b] W. A. Herrmann , C. Köcher , Angew. Chem. Int. Ed. Engl. 1997, 36, 2162;

[11c] D. Bourissou , O. Guerret , F. P. Gabbaï , G. Bertrand , Chem. Rev. 2000, 100, 39. 11749234 10.1021/cr940472u

[12] V. Nesterov , D. Reiter , P. Bag , P. Frisch , R. Holzner , A. Porzelt , S. Inoue , Chem. Rev. 2018, 118, 9678. 29969239 10.1021/acs.chemrev.8b00079

[13a] P. L. Arnold , S. T. Liddle , Chem. Commun. 2006, 3959; 10.1039/b606829d 17003868

[13b] P. de Frémont , N. Marion , S. P. Nolan , Coord. Chem. Rev. 2009, 253, 862;

[13c] F. E. Hahn , M. C. Jahnke , Angew. Chem. Int. Ed. 2008, 47, 3122; 10.1002/anie.200703883 18398856

[14a] W. A. Herrmann , Angew. Chem. Int. Ed. 2002, 41, 1290; 10.1002/1521-3773(20020415)41:8<1290::aid-anie1290>3.0.co;2-y 19750753

[14b] F. Glorius , N-Heterocyclic Carbenes in Transition Metal Catalysis, Springer, Berlin, Heidelberg, 2007;

[14c] M. N. Hopkinson , C. Richter , M. Schedler , F. Glorius , Nature 2014, 510, 485. 24965649 10.1038/nature13384

[15a] S. Richeter , A. Hadj-Aïssa , C. Taffin , A. van der Lee , D. Leclercq , Chem. Commun. 2007, 2148; 10.1039/b704681b 17520118

[15b] J.-F. Lefebvre , M. Lo , J.-P. Gisselbrecht , O. Coulembier , S. Clément , S. Richeter , Chem. Eur. J. 2013, 19, 15652; 24123635 10.1002/chem.201301483

[15c] J.-F. Longevial , A. Langlois , A. Buisson , C. H. Devillers , S. Clément , A. van der Lee , P. D. Harvey , S. Richeter , Organometallics 2016, 35, 663.

[16] M. Toganoh , T. Hihara , H. Furuta , Inorg. Chem. 2010, 49, 8182. 20726565 10.1021/ic1016356

[17a] J. C. Garrison , W. G. Kofron , R. S. Simons , C. A. Tessier , W. J. Youngs , US2004097723A1, 2001; 10.1039/b105751k 12240312

[17b] R. S. Simons , J. C. Garrison , W. G. Kofron , C. A. Tessier , W. J. Youngs , Tetrahedron Lett. 2002, 43, 3423.

[18] M. Moser, Ph.D. thesis, Ruprecht-Karl-Universität Heidelberg, Heidelberg, **2007**.

[19] K. Lin , L.-E. Chile , S. C. Zhen , P. D. Boyd , D. C. Ware , P. J. Brothers , Inorg. Chim. Acta 2014, 422, 95.

[20] M. Moser , B. Wucher , D. Kunz , F. Rominger , Organometallics 2007, 26, 1024.

[21] T. Hafner, PhD thesis, Ruprecht-Karl-Universität Heidelberg, Heidelberg, **2009**.

[22] J. Bouffard , B. K. Keitz , R. Tonner , V. Lavallo , G. Guisado-Barrios , G. Frenking , R. H. Grubbs , G. Bertrand , Organometallics 2011, 30, 2617. 21572542 10.1021/om200272m PMC3092707

[23] G. Guisado-Barrios , J. Bouffard , B. Donnadieu , G. Bertrand , Angew. Chem. Int. Ed. 2010, 49, 4759; 10.1002/anie.201001864 PMC3131155 20509134

[24] D. I. Bezuidenhout , G. Kleinhans , G. Guisado-Barrios , D. C. Liles , G. Ung , G. Bertrand , Chem. Commun. 2014, 50, 2431. 10.1039/c3cc49385g 24448261

[25a] A.-T. Schmidt, Diploma thesis, Eberhard Karls Universität Tübingen, Tübingen, **2013**;

[25b] A.-T. Schmidt, Ph.D. thesis, Eberhard Karls Universität Tübingen, Tübingen, **2018**.

[26a] Q. Wang , T. R. Chan , R. Hilgraf , V. V. Fokin , K. B. Sharpless , M. G. Finn , J. Am. Chem. Soc. 2003, 125, 3192; 12630856 10.1021/ja021381e

[26b] C. W. Tornøe , C. Christensen , M. Meldal , J. Org. Chem. 2002, 67, 3057; 11975567 10.1021/jo011148j

[26c] M. Meldal , C. W. Tornøe , Chem. Rev. 2008, 108, 2952. 18698735 10.1021/cr0783479

[27] H. C. Kolb , M. G. Finn , K. B. Sharpless , Angew. Chem. Int. Ed. 2001, 40, 2004; 10.1002/1521-3773(20010601)40:11<2004::AID-ANIE2004>3.0.CO;2-5 11433435

[28] H.-C. Gee , C.-H. Lee , Y.-H. Jeong , W.-D. Jang , Chem. Commun. 2011, 47, 11963. 10.1039/c1cc14963f 21969109

[29] I. Pryjomska-Ray , D. Zornik , M. Pätzel , K. B. Krause , L. Grubert , B. Braun-Cula , S. Hecht , C. Limberg , Chem. Eur. J. 2018, 24, 5341. 29265510 10.1002/chem.201704858

[30] D. Zornik , R. M. Meudtner , T. El Malah , C. M. Thiele , S. Hecht , Chem. Eur. J. 2011, 17, 1473. 21268150 10.1002/chem.201002491

[31] L. Arnold, PhD thesis, Johannes Gutenberg-Universität Mainz, Mainz, **2012**.

[32] Deposition numbers 2022047 (**5**), 2022049 (**6**) and 2022048 (**8**) contain the supplementary crystallographic data for this paper. These data are provided free of charge by the joint Cambridge Crystallographic Data Centre and Fachinformationszentrum Karlsruhe Access Structures service.

[33] B. K. Reddy , A. Basavarajappa , M. D. Ambhore , V. G. Anand , Chem. Rev. 2017, 117, 3420. 27966924 10.1021/acs.chemrev.6b00544

[34] C. Maeda , T. Yoneda , N. Aratani , M.-C. Yoon , J. M. Lim , D. Kim , N. Yoshioka , A. Osuka , Angew. Chem. Int. Ed. 2011, 50, 5691; 10.1002/anie.201101864 21567699

[35] C. Azarias , D. Jacquemin , J. Phys. Chem. A 2016, 120, 2824. 27076284 10.1021/acs.jpca.6b02313

[36] L. Arnold , H. Norouzi-Arasi , M. Wagner , V. Enkelmann , K. Müllen , Chem. Commun. 2011, 47, 970. 10.1039/c0cc03052j 21082110

[37] L. Arnold , M. Baumgarten , K. Müllen , Chem. Commun. 2012, 48, 9640. 10.1039/c2cc35550g 22908132

[38] C. Maeda , M. Masuda , N. Yoshioka , Org. Lett. 2013, 15, 3566. 23805949 10.1021/ol401403n

[39] C. Maeda , M. Masuda , N. Yoshioka , Org. Biomol. Chem. 2014, 12, 2656. 24618786 10.1039/c3ob42564a

[40] C. Maeda , K. Kurihara , M. Masuda , N. Yoshioka , Org. Biomol. Chem. 2015, 13, 11286. 26416478 10.1039/c5ob01824b

[41] C. Maeda , Y. Tanaka , T. Shirakawa , T. Ema , Chem. Commun. 2019, 55, 10162. 10.1039/c9cc05079e 31389426

[42] G. Calogero , G. Di Marco , S. Caramori , S. Cazzanti , R. Argazzi , C. A. Bignozzi , Energy Environ. Sci. 2009, 2, 1162.

[43] D. Tapu , D. A. Dixon , C. Roe , Chem. Rev. 2009, 109, 3385. 19281270 10.1021/cr800521g

[44] K. S. Flaig , B. Raible , V. Mormul , N. Denninger , C. Maichle-Mössmer , D. Kunz , Organometallics 2018, 37, 1291.

[45] D. Y. Dawson , J. Arnold , J. Porphyrins Phthalocyanines 1997, 1, 121.

[46] C. R. Groom , I. J. Bruno , M. P. Lightfoot , S. C. Ward , Acta Crystallogr. Sect. B 2016, 72, 171. 10.1107/S2052520616003954 PMC4822653 27048719

[47] A. S. de Sousa , M. A. Fernandes , W. Nxumalo , J. L. Balderson , T. Jeftič , I. Cukrowski , H. M. Marques , J. Mol. Struct. 2008, 872, 47.

[48] M. G. Sewchok , R. C. Haushalter , J. S. Merola , Inorg. Chim. Acta 1988, 144, 47.

[49a] J. Arnold , C. G. Hoffman , J. Am. Chem. Soc. 1990, 112, 8620;

[49b] J. Arnold , C. G. Hoffman , D. Y. Dawson , F. J. Hollander , Organometallics 1993, 12, 3645.

[50] D. Barisic , D. Diether , C. Maichle-Mössmer , R. Anwander , J. Am. Chem. Soc. 2019, 141, 13931. 31362492 10.1021/jacs.9b06879

[51a] P. Bougeard , M. Mancini , B. G. Sayer , M. J. McGlinchey , Inorg. Chem. 1985, 24, 93;

[51b] M. Mancini , P. Bougeard , R. C. Burns , M. Mlekuz , B. G. Sayer , J. I. A. Thompson , M. J. McGlinchey , Inorg. Chem. 1984, 23, 1072.

