# Supplementary material for: Carbenaporphyrins: No Longer Missing Ligands in N‐Heterocyclic Carbene Chemistry
Source: Angew Chem Int Ed Engl. 2020 Nov 27;60(4):2007–12. doi: 10.1002/anie.202013434 (PMC7898644; doi:10.1002/anie.202013434)
Supplement: Supplementary file 1 — Supplementary [file ANIE-60-2007-s001.pdf]

Angewandte Chemie
Eine Zeitschrift der Gesellschaft Deutscher Chemiker

Supporting Information

# Carbenaporphyrins: No Longer Missing Ligands in N-Heterocyclic Carbene Chemistry

*Theo Maulbetsch and Doris Kunz**

anie_202013434_sm_miscellaneous_information.pdf

Table of Contents

**1. General Information** ......... **2**

**2. Numbering Scheme** ......... **3**

**3. Experimental Details and Characterization** ......... **4**

**4. X-ray Crystal Structure Analysis** ......... **11**

**5. UV/Vis and Fluorescence Spectra** ......... **12**

*a) Experimental and calculated UV/Vis, and fluorescence spectra of **6** and **9**.* ......... 12

*b) Calculated UV/Vis spectra of **1** and **7**.* ......... 20

**6. NMR Spectra** ......... **26**

**7. DFT Calculations** ......... **35**

**8. References** ......... **40**

# 1. General Information

Unless otherwise stated, all reactions were carried out under an argon atmosphere in dried and degassed solvents using Schlenk technique. Toluene, tetrahydrofuran, dichloromethane and diethyl ether were purchased from Sigma Aldrich and dried using an MBraun SPS-800 solvent purification system. Deuterated solvents were dried with standard purification methods and degassed.[1] Chemicals from commercial suppliers were degassed through freeze-pump-thaw cycles prior to use. The precursors **2**[2] and **3**[3] were synthesized according to the literature. $^{1}$H and $^{13}$C NMR spectra were recorded using a Bruker AVANCE II+ 400 spectrometer, a Bruker AVANCE AVII+ 500 or a Bruker Avance III HDX 600 or a Bruker Avance III HDX 700. Chemical shifts δ (ppm) are given relative to the solvent's residual proton and carbon signal respectively: THF-$d_8$: 3.58 ppm ($^{1}$H NMR) and 67.57 ppm ($^{13}$C NMR); $CD_3CN$: 1.94 ppm ($^{1}$H NMR) and 1.32 ppm ($^{13}$C NMR); DMSO-$d_6$: 2.50 ppm ($^{1}$H NMR) and 39.51 ppm ($^{13}$C NMR), $CDCl_3$: 7.27 ppm ($^{1}$H NMR) and 77.0 ppm ($^{13}$C NMR), $CD_2Cl_2$: 5.32 ppm ($^{1}$H NMR) and 53.84 ppm ($^{13}$C NMR). Coupling constants (*J*) are expressed in Hz. Signals were assigned as s (singlet), d (doublet), t (triplet), q (quartet), m (multiplet) and variations thereof. Assignment of the peaks was made using 2D NMR correlation spectra. The mass spectra were recorded on a Bruker amazon SL mass spectrometer. UV/VIS spectra were measured using a Jasco V-770 UV-Visible/NIR spectrophotometer. Fluorescence spectra were recorded with a PTI Quantamaster QM4 spectrofluorometer equipped with a 75 W continuous xenon short arc lamp as excitation source. Spectral selection was achieved by a single grating monochromator with 1200 grooves/mm (300 nm blaze). The emission was monitored using a single grating monochromator at 1200 grooves/mm (500 nm blaze) and detected with the equipped standard detector module. Samples were prepared under inert atmosphere and measured in quartz fluorescence cuvettes (pathlength of 10 mm) with dry solvents. Solid state samples were measured under inert atmosphere in a glass tube. The elemental analysis was determined using a varioMICRO cube by the elemental analysis section of the Institut für Anorganische Chemie at the University of Tübingen and the high-resolution mass spectra were recorded using a Bruker Daltronics maXis 4G by the mass spectra section of the Institut für Organische Chemie at the University of Tübingen. The melting point was measured with a Büchi Melting Point M-560 apparatus.

*X-ray structure analysis.* Crystallographic data collection was carried out on a Bruker APEX Duo CCD with an Incoatec I*µ*S Microsource with a Quazar MX mirror using Mo $K_\alpha$ radiation ($\lambda$ = 0.71073 Å) or using Cu $K_\alpha$ radiation ($\lambda$ = 1.54056 Å) and a graphite monochromator. Corrections for absorption effects were applied using SADABS.[4] All structures were solved by direct methods using SHELXS and refined using SHELXL.[5] CCDC 2022047 (**5**), 2022048 (**8**) and 2022049 (**6**) contain the supplementary crystallographic data. These data can be obtained

free of charge from the Cambridge Crystallographic Data Centre at www.ccdc.cam.ac.uk/data_request/cif.

*DFT calculations*. All calculations were carried out using the ORCA[6] software package. The RI approximation was used for GGA calculations, whereas the RIJCOSX approximation was used for hybrid-DFT calculations.[7] All structures were optimized using the BP86 functional[8] and the def2-TZVP[7] basis set with convergence criteria for minimum energy change of $10^{-8}$ Hartree for the SCF part and $10^{-6}$ Hartree for the geometry optimization. TD-DFT[9] calculations were carried out using the *in vacuo* optimized geometry with the B3LYP functional and the def2-TZVP basis set with solvent effects being applied with the conductor-like polarizable continuum model for THF. The cartesian coordinates of the geometry optimized structures are provided as the supplementary file DFT_coordinates.xyz.

## 2. Numbering Scheme

For the assignment of the atoms in the X-ray molecular structure and the peaks in NMR spectra, the following numbering scheme was used:

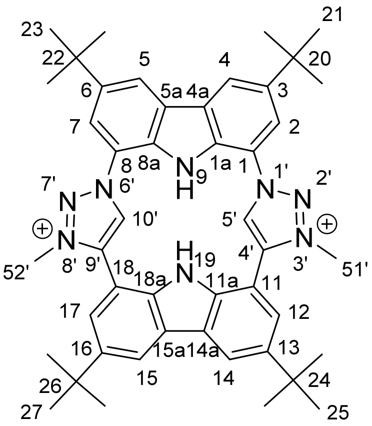

# 3. Experimental Details and Characterization

Synthesis of 1,8-Diazido-3,6-di-*tert*-butyl-carbazole (**4**)

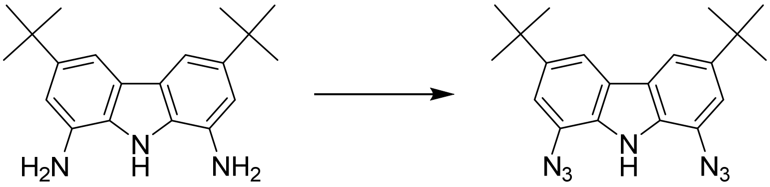

According to a general procedure,[10] 1,8-diamino-3,6-di-*tert*-butyl-carbazol (666 mg, 2.15 mmol, 1.0 eq) was dissolved in a mixture of 8 mL hydrochloric acid (37 %), 8 mL water and 50 mL ethanol. After cooling in an ice bath at 0 °C a solution of sodium nitrite (446 mg, 6.46 mmol, 3.0 eq) in 1 mL of water was added over a period of 10 min so that the temperature stayed below 5 °C. After stirring for 30 min a solution of sodium azide (420 mg, 6.46 mmol, 3.0 eq) in 2 mL of water was added slowly over a period of 10 min so that the temperature stayed below 5 °C. The reaction mixture was stirred for 1 h at 0 °C and another 2 h at room temperature. After addition of 75 mL of water and 150 mL of ethyl acetate the organic layer was separated, and the aqueous phase was extracted three times with 75 mL of ethyl acetate. The combined organic phases were washed two times each with 50 mL of saturated sodium hydrogen carbonate solution and 50 mL of brine. After drying over magnesium sulfate and removing the solvent *in vacuo*, the crude product was purified by means of column chromatography (silica; dichloromethane:petroleum ether (40/60) 1:3) yielding 722 mg (93 %) of product **4** as a light yellow, light sensitive solid.

**$^1$H NMR** (DMSO-d$_6$, 400.11 MHz): δ 11.15 (s, 1H, H-9), 8.03 (d, $^4J_{HH}$ = 1.4 Hz, 2H, H-4/5 or H-2/7), 7.28 (d, $^4J_{HH}$ = 1.4 Hz, 2H, H-4/5 or H-2/7), 1.41 (s, 18H, C(CH$_3$)$_3$).

**$^1$H NMR** (CD$_2$Cl$_2$, 400.11 MHz): δ 8.16 (s, 1H, H-9), 7.86 (d, $^4J_{HH}$ = 1.5 Hz, 2H, H-4/5), 7.28 (d, $^4J_{HH}$ = 1.5 Hz, 2H, H-2/7), 1.45 (s, 18H, C(CH$_3$)$_3$).

**$^{13}$C NMR** (DMSO-d$_6$, 100.61 MHz): δ 143.0 (C3/6), 130.3, 124.5, 122.8 (C1/8, C1a/8a, C4a/5a), 113.85, 113.0 (C2/7, C4/5), 34.7 (*C*(CH$_3$)$_3$), 31.7 (C(*C*H$_3$)$_3$).

Synthesis of macrocycle **5**

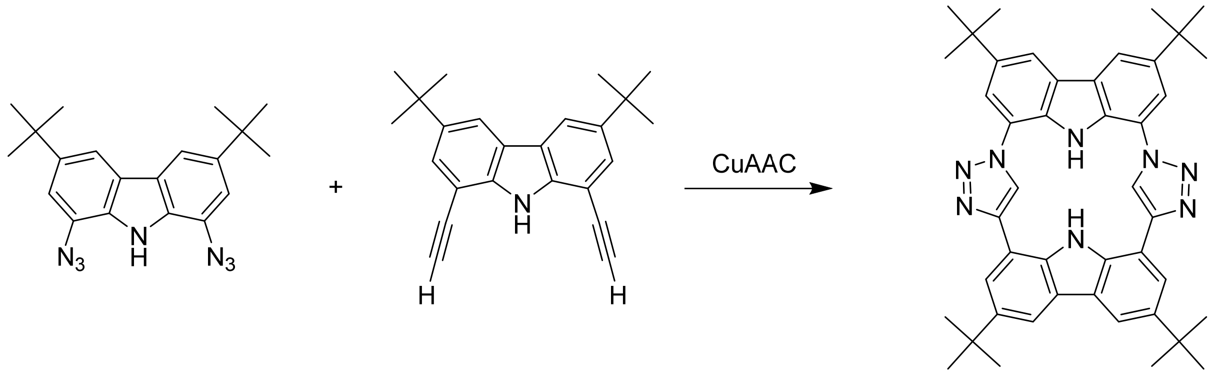


In a modified procedure,[11] a round bottom flask with copper sulfate pentahydrate (6.0 mg, 22 *µ*mol, 0.1 eq), TBTA (12 mg, 22 *µ*mol, 0.1 eq) and sodium ascorbate (1.2 mg, 66 *µ*mol, 0.3 eq) were put under argon. 0.5 mL of triethyl amine and 30 mL of degassed tetrahydrofuran were added. The flask was covered with a septum and heated to 60 °C. Then a solution of 1,8 -diazido-3,6-di-*tert*-butyl-carbazole (80.0 mg, 220 *µ*mol, 1 eq) and 1,8 -diethinyl-3,6-di-*tert*-butyl-carbazole (72.0 mg, 220 *µ*mol, 1 eq) in 10 mL degassed tetrahydrofuran was added with a syringe pump at 10 *µ*L/min. After completion, the reaction was stirred at 60 °C for 3 h. After concentration of the suspension *in vacuo* to 2 mL, 5 mL of methanol were added and the precipitate was filtered off and washed with 1 mL of methanol to yield 79.0 mg (52 %) of the product **5** as a white solid.

**$^1$H NMR** (THF-d$_8$, 500.11 MHz): δ 10.19 (s, 1H, H-9), 9.79 (s, 2H, H-5'/10), 9.43 (s, 1H, H-19), 8.41 (d, $^4J_{HH}$=1.7 Hz, 2H, H-2/7 or H-4/5), 8.24 (d, $^4J_{HH}$ = 1.9 Hz, 2H, H-14/15), 8.19 (d, $^4J_{HH}$ = 1.7 Hz, 2H, H-2/7 or H-4/5), 8.10 (d, $^4J_{HH}$ = 1.9 Hz, 2H, H-12/17), 1.57 (s, 18H, H-25/27), 1.55 (s, 18H, H-21/23).

**$^{13}$C NMR** (THF-d$_8$, 125.11 MHz): δ 146.53, 146.48 (C3/6, C4'/9'), 144.7 (C13/16), 139.4 (C14a/15a), 133.4 (C4a/5a), 128.6 (C1/C8), 126.8 (C11/18), 124.8 (C5'/10'), 124.3 (C1a/8a), 122.6 (C12/17), 118.9, 118.5, 118.3 (C2/7, C4/5, C14/15), 116.1 (C11a/18a), 36.1 (C20/22), 35.8 (C24/26), 32.6 (C25/27), 32.4 (C21/23).

Synthesis of macrocycle **6**

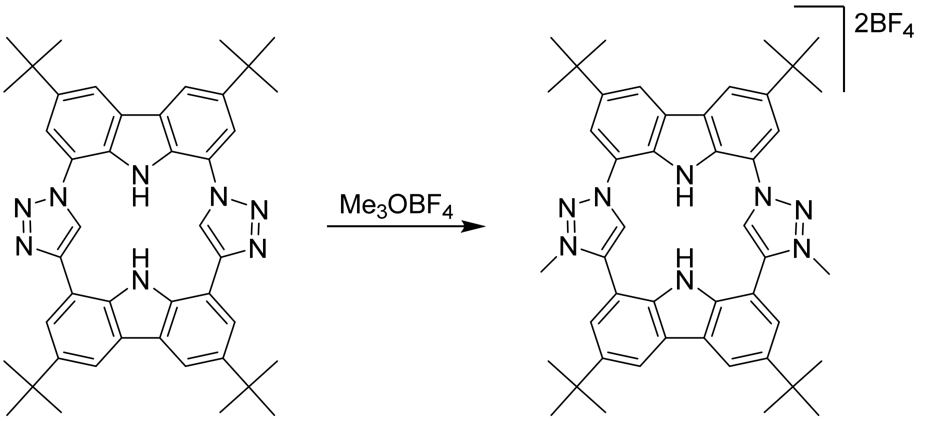


A suspension of macrocycle **5** (100 mg, 145 *μ*mol, 1 eq) and trimethyloxonium tetrafluoroborate (42.9 mg, 290 *μ*mol, 2 eq) in 20 mL of dichloromethane was stirred over night at room temperature. After concentration *in vacuo* to about 1 mL, 25 mL of diethyl ether were added and the precipitate was filtered off and washed twice with 5 mL diethyl ether to give 129 mg (99 %) of product **6** as an off-white solid.

**$^{1}$H NMR** (THF-$d_8$, 400.11 MHz): δ 10.01 (br s, 2H). 9.77 (br s, 2H), 8.60 (br s, 2H), 8.51 (br s, 2H), 8.07 (br s, 2H), 7.99 (br s, 2H), 4.55 (s, 6H), 1.53 (br s, 36H).

**$^{1}$H NMR** ($CD_3CN$, 400.11 MHz): δ 9.58 (br s, 2H), 9.51 (s, 2H), 8.67 (s, 2H), 8.62 (d, $^{4}J_{HH}$ = 1.3 Hz, 2H), 8.07 (s, 2H), 7.94 (d, $^{4}J_{HH}$ = 1.3 Hz, 2H), 4.52 (s, 6H), 1.56 (s, 18H), 1.55 (s, 18H).

**$^{1}$H NMR** ($CD_2Cl_2$, 400.11 MHz): δ 9.92 (s, 2H, H-5'/10'), 9.40 (br s, 2H, H-9, H-19), 8.48 (d, $^{4}J_{HH}$ = 1.3 Hz, 2H, H-4/5), 8.47 (d, $^{4}J_{HH}$ = 1.4 Hz, 2H, H-14/15), 7.97 (d, $^{4}J_{HH}$ = 1.5 Hz, 2H, H-2/7), 7.73 (d, $^{4}J_{HH}$ = 1.5 Hz, 2H, H-12/17), 4.51 (s, 6H, Me), 1.56 (s, 18H, H-25/27), 1.55 (s, 18H, H-21/23).

**$^{13}$C-NMR** ($CD_2Cl_2$, 100.61 MHz): δ 146.8 (C3/6), 145.4 (C13/16), 142.1 (C4'/9'), 140.0 (C11a/18a), 134.7 (C1/8 or C1a/8a or C4a/5a), 132.2 (C5'/10'), 128.3 (C1/8 or C1a/8a or C4a/5a), 126.3 (C14a/C15a), 124.3 (C12/17), 122.5, 122.4 (C4/5, C14/15), 121.0 (C1/8 or C1a/8a or C4a/5a), 119.7 (C2/7), 106.1 (C11/18), 39.2 (Me), 35.9, 35.7 ($\mathit{C}(CH_3)_3$), 32.2, 32.1 ($C(\mathit{C}H_3)_3$).

**HR-ESI$^{+}$** (MeCN): $[C_{46}H_{54}N_8]^{2+}$ m/z = 359.22361 – calculated: 359.22302.

**m.p.:** 340 °C (dec.)

Synthesis of the dilithium complex **7**

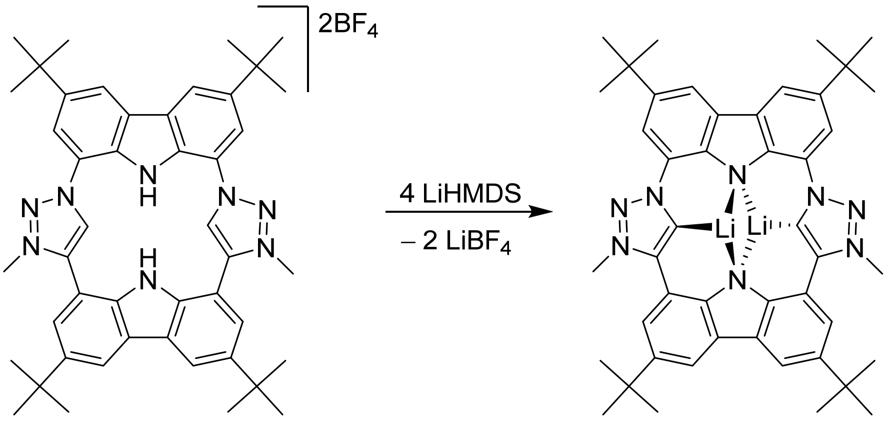


A solution of lithium bis(trimethylsilyl)amide (6.7 mg, 40 *µ*mol, 4 eq) in 0.3 mL tetrahydrofuran-d8 was added to a suspension of macrocycle **6** (9.0 mg, 10 *µ*mol, 1 eq) in 0.2 mL tetrahydrofuran-d8 to give the product **7** in 99 % yield (NMR) as a yellow solution.

**$^1$H NMR** (THF-d8, 300.11 MHz): δ 8.22 (d, $^4J_{HH}$ = 2.0 Hz, 2H, H-4/5 or H-14/15), 8.23 (d, $^4J_{HH}$ = 1.8 Hz, 2H, H-4/5 or H-14/15), 8.04 (d, $^4J_{HH}$ = 1.8 Hz, 2H, H-2/7), 7.69 (d, $^4J_{HH}$ = 2.0 Hz, 2H, H-12/17), 4.48 (s, 6H, Me), 1.57 (s, 18H, $C(CH_3)_3$), 1.56 (s, 18H, $C(CH_3)_3$).

**$^{13}$C NMR** (THF-d8, 125.11 MHz): δ 187.9 (C5'/C10'), 151.7 (C11a/18a), 149.2 (C4'/C9'), 146.0 (C1a/C8a), 137.2 (C3/6), 136.1 (C13/16), 129.5, 128.7, 128.0 (C1/8 or C4a/5a or C11/C18 or C14a/15a), 120.5 (C12/17), 117.0, 116.8 (C4/5, C14/15), 116.1 (C1/8 or C4a/5a or C11/C18 or C14a/15a), 113.7 (C2/7), 37.3 (Me), 35.5 (C20/22), 35.4 (C24/26), 33.0 (C21/23 + C25/27).

**$^7$Li NMR** (THF-d8, 116.6 MHz): δ 0.57 (br s).

**$^7$Li NMR** (THF-d8, 116.6 MHz, −80 °C): δ 1.90 (br s).

Synthesis of complex **8**

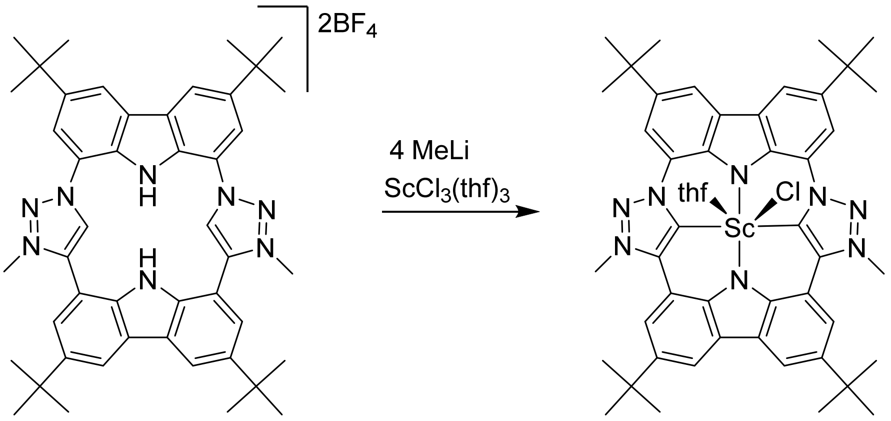


After *in situ* deprotonation of macrocycle **6** (8.9 mg, 10 *μ*mol) in 0.5 mL of tetrahydrofuran-d8 with methyl lithium (1.0 mg, 45 *μ*mol), [ScCl3(thf)3] (3.6 mg, 10 *μ*mol) was added to obtain the product **8** quantitatively (NMR) as an orange solution, which shows an orange fluorescence (excitation at *λ* = 366 nm).

**$^1$H NMR** (THF-d8, 400.11 MHz): δ 8.32 (s, 4H, H-4/5 + H-12/17 or H-14/15), 8.28 (d, $^4J_{HH}$ = 1.7 Hz, 2H, H-12/17 or H-14/15), 7.98 (d, $^4J_{HH}$ = 1.7 Hz, 2H, H-2/7), 4.64 (s, 6H, Me), 1.59 (s, 18H, C(CH3)3), 1.57 (s, 18H, C(CH3)3).

**$^{13}$C NMR** (THF-d8, 125.76 MHz): δ 148.0 (C1a/8a), 145.7 (C12/17), 141.6 (C11a/18a), 140.2, 139.2 (C3/6 and C13/16), 129.0, 127.5 (C4a/5a and C14a/15a), 126.5 (C11/18), 120.5 (C2/7), 117.8, 117.7 (C4/5 and C14/15), 114.4 (C1/8), 113.1 (C12/17), 38.8 (Me), 35.3, 35.2 (*C*(CH3)3), 32.4(C(*C*H3)3).

**$^{45}$Sc NMR** (121.5 MHz, THF-d8): δ 285.0.

Synthesis of complex **9**

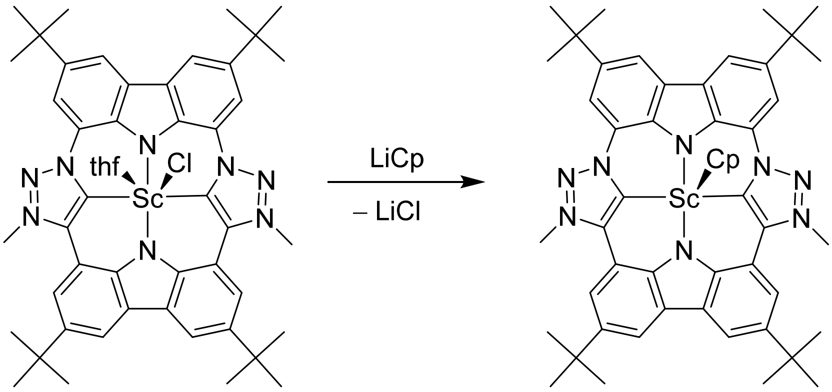


Lithium cyclopentadienide (1.6 mg, 22 *μ*mol) was added to an *in situ* prepared solution of complex **8** (from **6** (20 mg, 22 *μ*mol) and LiHMDS (15 mg, 90 *μ*mol) in 1.5 mL tetrahydrofuran). After removing all volatiles *in vacuo,* the solid was extracted two times with 1.5 mL toluene, and the combined extracts were dried *in vacuo*. The resulting orange residue was washed with pentane (2x 1.5 mL) and yielded 14.6 mg (80%) of complex **9** after drying *in vacuo* as an orange solid.

**$^1$H NMR** (THF-d$_8$, 400.11 MHz): δ 8.36 (d, $^4J_{HH}$ = 1.9 Hz, 2H, H-4/5), 8.35 (d, $^4J_{HH}$ = 1.8 Hz, 2H, H-12/17 or H-14/15), 8.33 (d, $^4J_{HH}$ = 1.8 Hz, 2H, H-12/17 or H-14/15), 8.03 (d, $^4J_{HH}$ = 1.9 Hz, 2H, H-2/7), 5.21 (s, 5H, Cp), 4.66 (s, 6H, Me), 1.61 (s, 18H, C(CH$_3$)$_3$), 1.59 (s, 18H, C(CH$_3$)$_3$).

**$^1$H NMR** (C$_6$D$_6$, 400.11 MHz): δ 8.75 (d, $^4J_{HH}$ = 1.8 Hz, H-14/15, 2H), 8.71 (d, $^4J_{HH}$ = 1.8 Hz, H-2/7 or H-4/5, 2 H), 8.68 (d, $^4J_{HH}$ = 1.8 Hz, H-2/7 or H-4/5, 2H), 7.64 (d, $^4J_{HH}$ = 1.8 Hz, H-12/17, 2H), 5.69 (s, 5H, Cp), 3.34 (s, 6H, Me), 1.61 (s, 18H, C(CH$_3$)$_3$),1.60 (s, 18H, C(CH$_3$)$_3$).

**$^{13}$C NMR** (THF-d$_8$, 125.76 MHz): δ 147.1 (C1a/8a), 145.3 (C12/17), 140.8 (C11a/18a), 140.4, 139.2 (C3/6, C13/16), 129.4 (C14a/15a), 127.9 (C4a/5a), 126.5 (C11/C18),

120.5 (C2/7), 118.0 (C4/5, C12/17 or C14/15), 114.2 (C1/8), 113.3 (C12/17 or C14/15), 110.4 (Cp), 38.9 (Me), 35.4, 35.3 (*C*$(CH_3)_3$), 32.4 (C(*C*$H_3)_3$).

**$^{45}$Sc NMR** (121.5 MHz, THF-$d_8$): δ 132.0.

**HR-ESI$^+$** (MeCN): $[C_{51}H_{56}N_8Sc]^+$ m/z = 825.41709 – calculated: 825.41816.

**EA:** Calcd for $C_{51}H_{55}N_8Sc$*0.33 $LiBF_4$: C, 71.54; N, 13.09; H, 6.47; found: C, 71.53; N, 12.91; H, 6.52.

# 4. X-ray Crystal Structure Analysis

**Table S1.** Crystallographic data and structure refinement for compounds **5**, **6**, and **8**.

| Compound | **5** | **6** | **8** |
|---|---|---|---|
| CCDC | 2022047 | 2022049 | 2022048 |
| Empirical formula | $C_{62}H_{84}N_8O_{4.50}$ | $C_{58}H_{78}B_2F_8N_8O_3$ | $C_{50}H_{58}ClN_8OSc$ |
| Formula weight | 1013.37 | 1108.90 | 867.45 |
| Temperature | 100(2) K | 100(2) K | 100(2) K |
| Radiation wavelength | MoK$_{\alpha}$ ($\lambda$ = 0.71073 Å) | CuK$_{\alpha}$ ($\lambda$ = 1.54178 Å) | MoK$_{\alpha}$ ($\lambda$ = 0.71073 Å) |
| Crystal system | Triclinic | Triclinic | Triclinic |
| Space group | P-1 | P-1 | P-1 |
| Unit cell dimensions | a = 13.358(2) Å | a = 13.3579(5) Å | a = 10.5932(3) Å |
| | b = 13.426(2) Å | b = 13.5935(5) Å | b = 15.9956(5) Å |
| | c = 15.830(3) Å | c = 18.6723(8) Å | c = 16.5273(5) Å |
| | α = 78.382(3)° | α = 98.686(3)° | α = 88.4420(10)° |
| | β = 85.129(3)° | β = 95.781(3)° | β = 72.5520(10)° |
| | γ = 88.732(3)° | γ = 95.576(3)° | γ = 88.0000(10)° |
| Volume | 2770.8(8) Å$^3$ | 3312.8(2) Å$^3$ | 2669.57(14) Å$^3$ |
| Z | 2 | 2 | 2 |
| Density (calculated) | 1.215 Mg/m$^3$ | 1.112 Mg/m$^3$ | 1.079 Mg/m$^3$ |
| Absorption coefficient | 0.077 mm$^{-1}$ | 0.702 mm$^{-1}$ | 0.228 mm$^{-1}$ |
| F(000) | 1096 | 1176 | 920 |
| Crystal size | 0.277 x 0.198 x 0.122 mm$^3$ | 0.243 x 0.159 x 0.128 mm$^3$ | 0.273 x 0.155 x 0.101 mm$^3$ |
| Theta range for data collection | 1.318 to 28.322°. | 2.410 to 69.240°. | 1.292 to 30.333°. |
| Index ranges | -17<=h<=17, -17<=k<=17, -21<=l<=21 | -16<=h<=16, -16<=k<=16, -22<=l<=22 | -15<=h<=15, -22<=k<=22, -23<=l<=23 |
| Reflections collected | 66037 | 65562 | 142739 |
| Independent reflections | 13775 [R(int) = 0.0810] | 11840 [R(int) = 0.1213] | 15990 [R(int) = 0.0576] |
| Completeness to theta = 25.242° | 100.0 % | 96.5 % | 99.9 % |
| Absorption correction | Numerical | Numerical | Numerical |
| Data / restraints / parameters | 13775 / 162 / 751 | 11840 / 310 / 840 | 15990 / 66 / 628 |
| Goodness-of-fit on F2 | 1.031 | 1.037 | 1.045 |
| Final R indices [I>2sigma(I)] | R1 = 0.0596, wR2 = 0.1487 | R1 = 0.0818, wR2 = 0.2196 | R1 = 0.0591, wR2 = 0.1712 |
| R indices (all data) | R1 = 0.0931, wR2 = 0.1715 | R1 = 0.1155, wR2 = 0.2505 | R1 = 0.0758, wR2 = 0.1859 |
| Largest diff. peak and hole | 0.732 and -0.628 e·Å$^{-3}$ | 0.876 and -0.637 e·Å$^{-3}$ | 0.667 and -0.798 e·Å$^{-3}$ |

# 5. UV/Vis and Fluorescence Spectra

## *a) Experimental and calculated UV/Vis, and fluorescence spectra of **6** and **9**.*

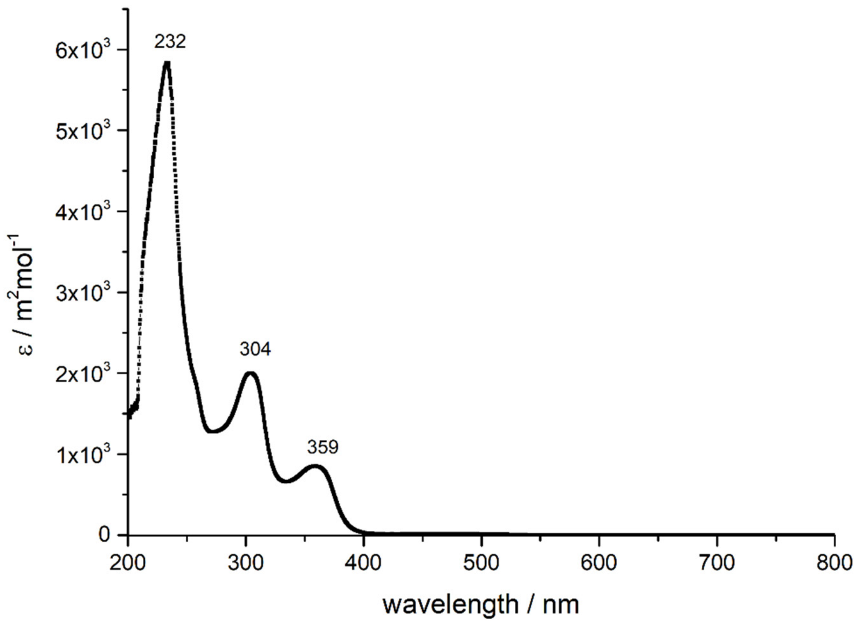


**Figure S1.** Absorption spectrum of **6** (tetrahydrofuran, c = 6*10$^{-5}$ M).

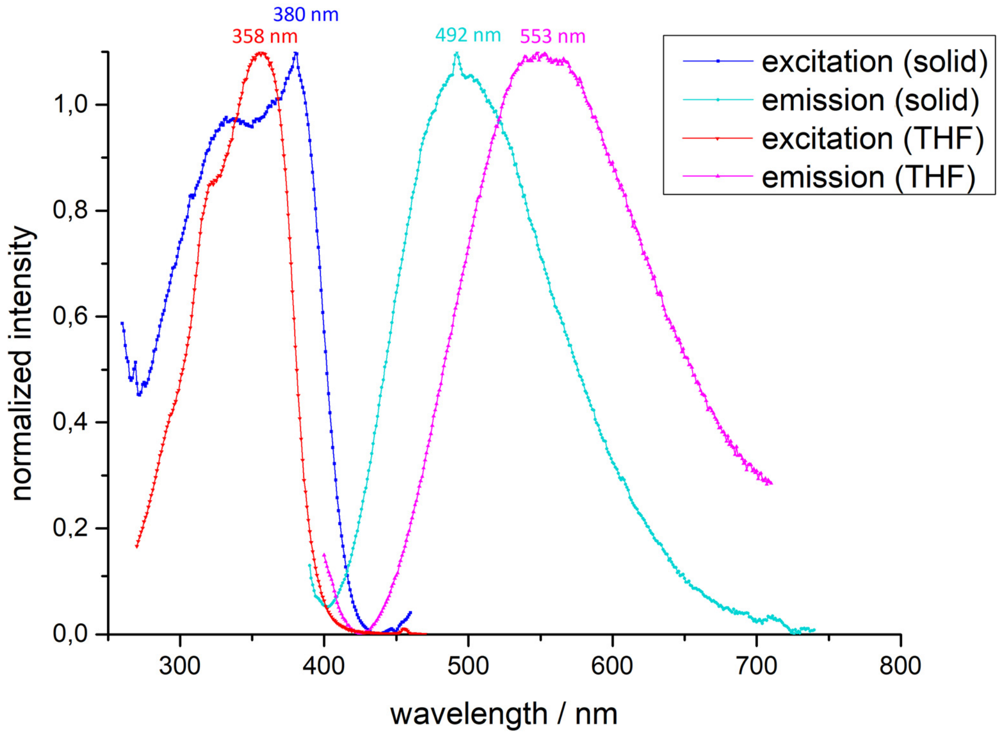


**Figure S2.** Excitation and emission spectra of **6** in the solid and in solution (tetrahydrofuran, c = 5*10$^{-5}$ M).

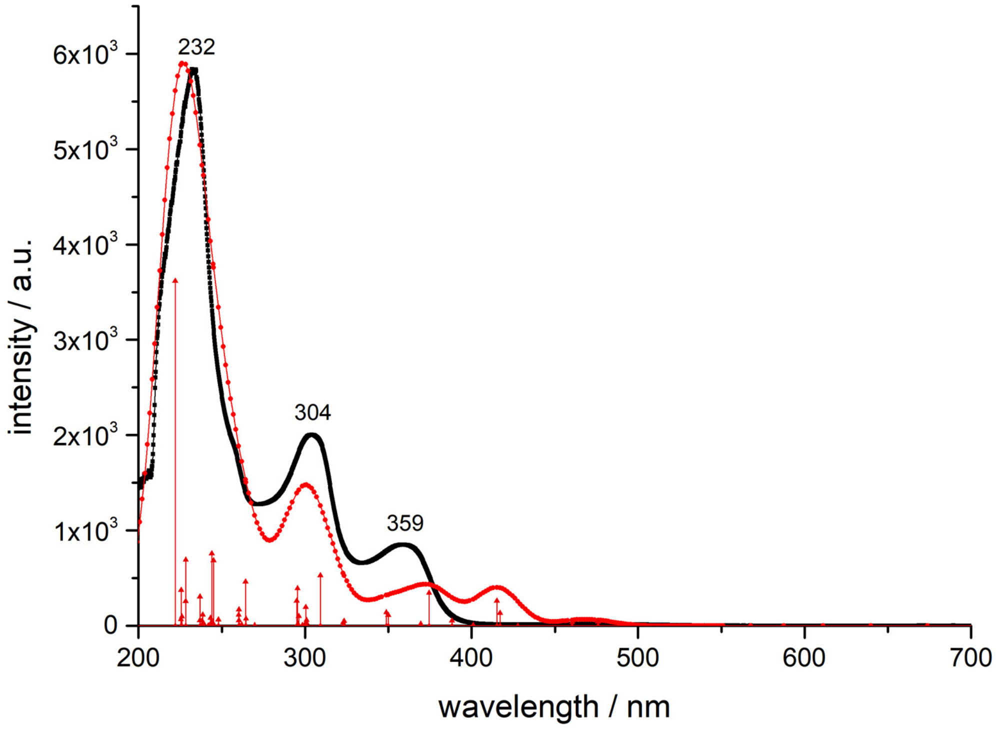


**Figure S3.** Comparison of the measured (black) and the calculated (red) absorption spectrum of **6** (calculated with TD-DFT B3LYP/def2-TZVP, CPCM(thf)).

**Table S2.** Calculated states of the absorption spectrum of compound **6** via transition electric dipole moments (TD-DFT: B3LYP/def2-TZVP, RIJCOSX, CPCM (thf)).

| State | Energy | Wavelength | $f_{osc}$ | $T^2$ | $T_X$ | $T_Y$ | $T_Z$ |
|---|---|---|---|---|---|---|---|
| | (cm$^{-1}$) | (nm) | | (a.u.$^2$) | (a.u.) | (a.u.) | (a.u.) |
| **1** | 21003.0 | 476.1 | 0.01384278 | 0.21698 | -0.02016 | -0.46490 | 0.02097 |
| **2** | 21717.4 | 460.5 | 0.01337773 | 0.20279 | -0.44678 | -0.03877 | 0.04098 |
| **3** | 24086.6 | 415.2 | 0.08630565 | 1.17961 | -0.39016 | -0.89879 | -0.46859 |
| **4** | 23978.6 | 417.0 | 0.04409531 | 0.60540 | -0.68311 | 0.33272 | 0.16751 |
| **5** | 23377.0 | 427.8 | 0.00028333 | 0.00399 | -0.01463 | -0.06145 | -0.00047 |
| **6** | 24940.4 | 401.0 | 0.00211681 | 0.02794 | 0.09492 | 0.10469 | 0.08929 |
| **7** | 25759.6 | 388.2 | 0.01795857 | 0.22951 | -0.42121 | 0.20748 | 0.09513 |
| **8** | 26705.6 | 374.5 | 0.11444217 | 1.41078 | -0.31568 | -1.05558 | -0.44372 |
| **9** | 28669.9 | 348.8 | 0.04711680 | 0.54104 | -0.10590 | -0.72694 | -0.03704 |
| **10** | 27064.1 | 369.5 | 0.00696615 | 0.08474 | -0.09590 | -0.25146 | -0.11095 |
| **11** | 30906.0 | 323.6 | 0.01643865 | 0.17511 | 0.41728 | -0.02172 | -0.02261 |
| **12** | 32340.8 | 309.2 | 0.17583648 | 1.78992 | -1.30725 | 0.25990 | 0.11610 |
| **13** | 28572.8 | 350.0 | 0.03699546 | 0.42626 | -0.63693 | 0.13268 | 0.05451 |

| | | | | | | | |
|---|---|---|---|---|---|---|---|
| **14** | 33228.7 | 300.9 | 0.02066903 | 0.20478 | 0.44908 | -0.05427 | -0.01269 |
| **15** | 30966.5 | 322.9 | 0.01183935 | 0.12587 | -0.15731 | -0.31722 | -0.02218 |
| **16** | 33359.1 | 299.8 | 0.00430687 | 0.04250 | 0.19214 | -0.06337 | -0.03961 |
| **17** | 33814.2 | 295.7 | 0.00706818 | 0.06882 | -0.06891 | -0.16753 | -0.18974 |
| **18** | 33236.9 | 300.9 | 0.01090334 | 0.10800 | 0.08367 | 0.29379 | -0.12117 |
| **19** | 33897.8 | 295.0 | 0.08675355 | 0.84254 | -0.23486 | -0.87144 | -0.16728 |
| **20** | 33762.0 | 296.2 | 0.03318690 | 0.32360 | 0.55518 | -0.11748 | -0.03976 |
| **21** | 33285.2 | 300.4 | 0.06488933 | 0.64180 | 0.16996 | 0.70045 | 0.34969 |
| **22** | 33838.9 | 295.5 | 0.13067945 | 1.27135 | -0.23671 | -0.99638 | -0.47175 |
| **23** | 33505.0 | 298.5 | 0.00254185 | 0.02498 | 0.15318 | -0.03464 | -0.01769 |
| **24** | 37845.5 | 264.2 | 0.15302369 | 1.33113 | 1.12549 | -0.23327 | -0.09992 |
| **25** | 38424.8 | 260.2 | 0.05712403 | 0.48942 | -0.68560 | 0.12795 | 0.05485 |
| **26** | 38171.5 | 262.0 | 0.00307970 | 0.02656 | -0.15263 | 0.05580 | -0.01230 |
| **27** | 38411.6 | 260.3 | 0.01848100 | 0.15839 | 0.07807 | 0.13684 | 0.36548 |
| **28** | 37788.9 | 264.6 | 0.02521872 | 0.21970 | -0.45905 | 0.08634 | 0.03895 |
| **29** | 38432.8 | 260.2 | 0.03829531 | 0.32803 | 0.55492 | -0.12839 | -0.06009 |
| **30** | 37066.8 | 269.8 | 0.00236965 | 0.02105 | 0.00932 | -0.13437 | 0.05388 |
| **31** | 40839.7 | 244.9 | 0.00792963 | 0.06392 | -0.20957 | -0.14094 | -0.01179 |
| **32** | 40989.8 | 244.0 | 0.25123572 | 2.01781 | 0.27468 | 1.36166 | 0.29708 |
| **33** | 41343.4 | 241.9 | 0.00106222 | 0.00846 | 0.09039 | -0.00017 | -0.01698 |
| **34** | 40801.8 | 245.1 | 0.22749408 | 1.83555 | 0.33864 | 1.20526 | 0.51790 |
| **35** | 40336.9 | 247.9 | 0.02151662 | 0.17561 | -0.41698 | 0.03777 | 0.01763 |
| **36** | 38382.8 | 260.5 | 0.00094831 | 0.00813 | -0.03751 | -0.06994 | -0.04284 |
| **37** | 42221.7 | 236.8 | 0.10134556 | 0.79021 | 0.81045 | -0.34038 | -0.13237 |
| **38** | 41972.6 | 238.3 | 0.01736358 | 0.13619 | 0.36441 | 0.05657 | 0.01407 |
| **39** | 41917.5 | 238.6 | 0.03861064 | 0.30324 | 0.13227 | 0.50136 | 0.18544 |
| **40** | 41101.9 | 243.3 | 0.02773430 | 0.22214 | 0.42286 | -0.19540 | -0.07177 |
| **41** | 43781.6 | 228.4 | 0.23029750 | 1.73170 | 0.42452 | 1.19238 | 0.36016 |
| **42** | 43789.0 | 228.4 | 0.08613536 | 0.64758 | -0.28934 | -0.73385 | -0.15911 |
| **43** | 44346.8 | 225.5 | 0.02277526 | 0.16907 | 0.31736 | 0.25270 | 0.06708 |
| **44** | 40978.5 | 244.0 | 0.01391179 | 0.11176 | 0.26564 | 0.20273 | 0.01001 |
| **45** | 42221.1 | 236.8 | 0.01960128 | 0.15284 | 0.35591 | -0.14341 | -0.07485 |
| **46** | 45034.3 | 222.1 | 1.19912856 | 8.76594 | -2.85524 | 0.72639 | 0.29305 |
| **47** | 42019.9 | 238.0 | 0.00208797 | 0.01636 | 0.10470 | -0.07121 | -0.01803 |
| **48** | 44226.3 | 226.1 | 0.03285925 | 0.24460 | -0.47914 | -0.12253 | 0.00234 |
| **49** | 44318.0 | 225.6 | 0.12466355 | 0.92605 | -0.03687 | -0.92347 | -0.26813 |
| **50** | 41850.3 | 238.9 | 0.00935810 | 0.07361 | -0.01596 | -0.26259 | -0.06637 |

**Table S3.** Orbital contribution to the first 10 excited states (singlets; HOMO = 191; LUMO = 192) in compound **6**.

| State | The weight of the individual excitations (printed if larger than $10^{-02}$) |
|---|---|
| State 1 | 190a -> 192a : 0.020308 (c= -0.14250505)<br>191a -> 192a : 0.948251 (c= -0.97378158)<br>191a -> 193a : 0.013213 (c= 0.11494996) |
| State 2 | 190a -> 193a : 0.102587 (c= -0.32029249)<br>191a -> 192a : 0.013116 (c= -0.11452432)<br>191a -> 193a : 0.867916 (c= -0.93161984) |
| State 3 | 188a -> 193a : 0.029493 (c= -0.17173637)<br>190a -> 192a : 0.907056 (c= -0.95239476)<br>190a -> 193a : 0.015373 (c= 0.12398619)<br>191a -> 192a : 0.016272 (c= 0.12755996) |
| State 4 | 190a -> 192a : 0.016225 (c= 0.12737563)<br>190a -> 193a : 0.840674 (c= 0.91688252)<br>191a -> 193a : 0.096729 (c= -0.31101215)<br>191a -> 195a : 0.010018 (c= -0.10009046) |
| State 5 | 189a -> 192a : 0.969575 (c= 0.98467004) |
| State 6 | 189a -> 193a : 0.824055 (c= -0.90777463)<br>191a -> 194a : 0.148828 (c= 0.38578229) |
| State 7 | 188a -> 192a : 0.929377 (c= 0.96404203)<br>188a -> 196a : 0.022191 (c= -0.14896728) |
| State 8 | 188a -> 193a : 0.792993 (c= 0.89050177)<br>190a -> 192a : 0.018915 (c= -0.13753151)<br>191a -> 194a : 0.138492 (c= -0.37214503) |
| State 9 | 188a -> 193a : 0.136284 (c= -0.36916610)<br>189a -> 193a : 0.148993 (c= -0.38599568)<br>189a -> 195a : 0.016680 (c= -0.12914919)<br>189a -> 200a : 0.010315 (c= -0.10156449)<br>190a -> 192a : 0.011785 (c= 0.10855971)<br>191a -> 194a : 0.627826 (c= -0.79235441) |
| State 10 | 190a -> 194a : 0.965211 (c= 0.98245176) |

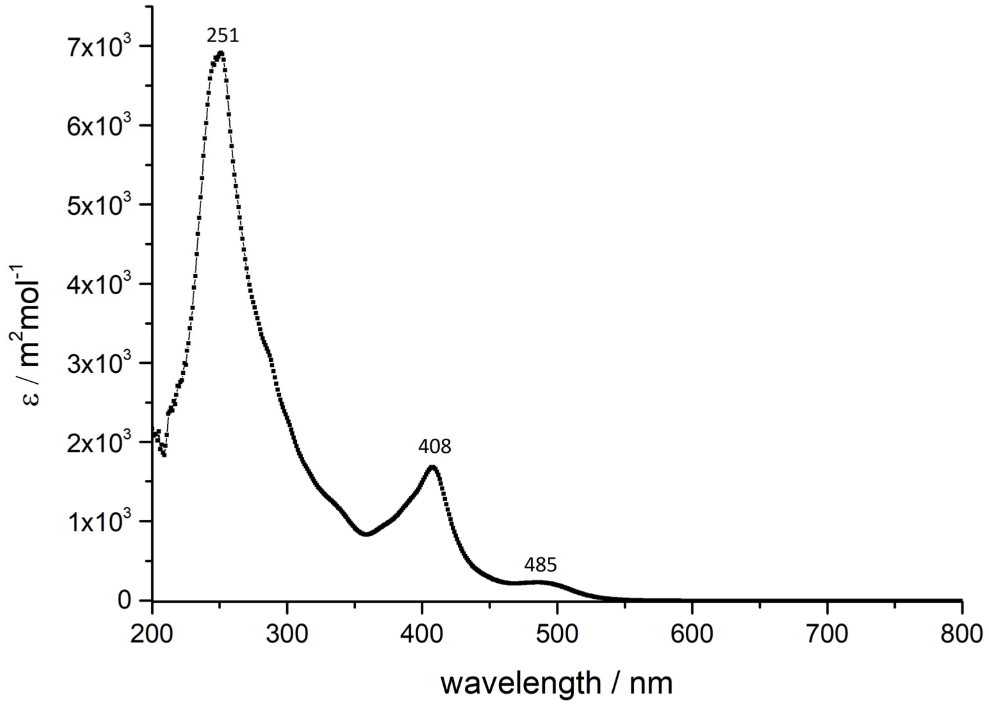


**Figure S4.** Absorption spectrum of complex **9** (tetrahydrofuran, c = 5*10$^{-5}$ M).

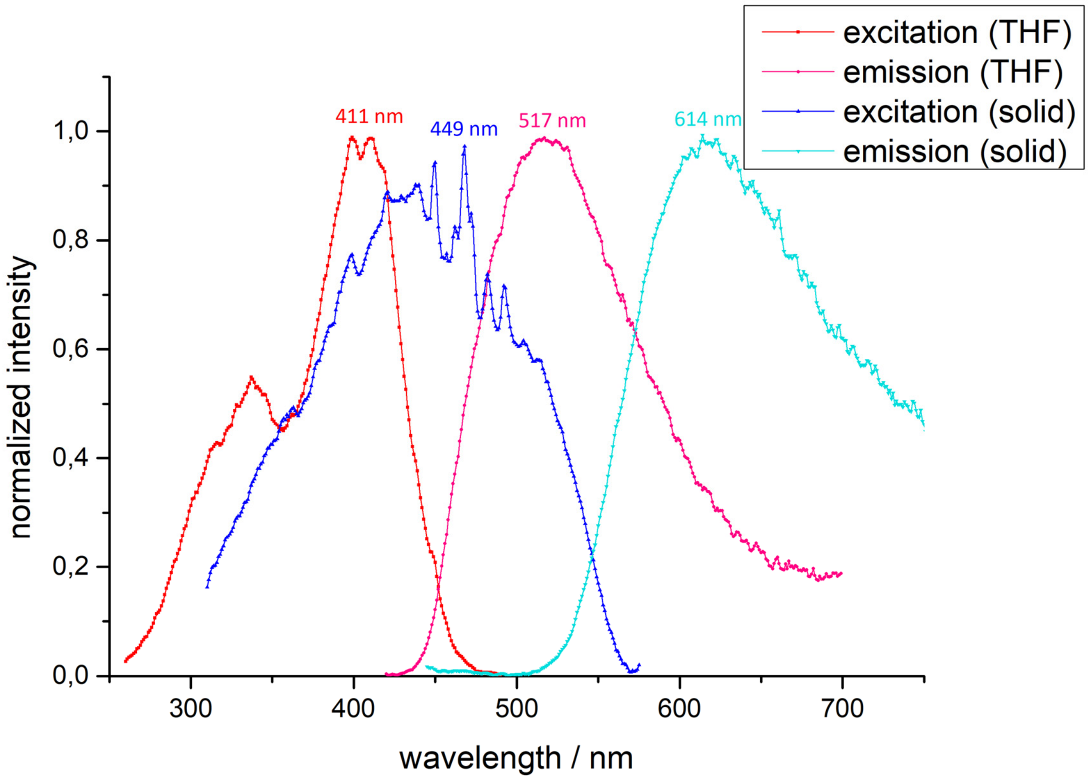


**Figure S5.** Excitation and emission spectra of complex **9** in the solid and in solution (tetrahydrofuran, c = 5*10$^{-5}$ M).

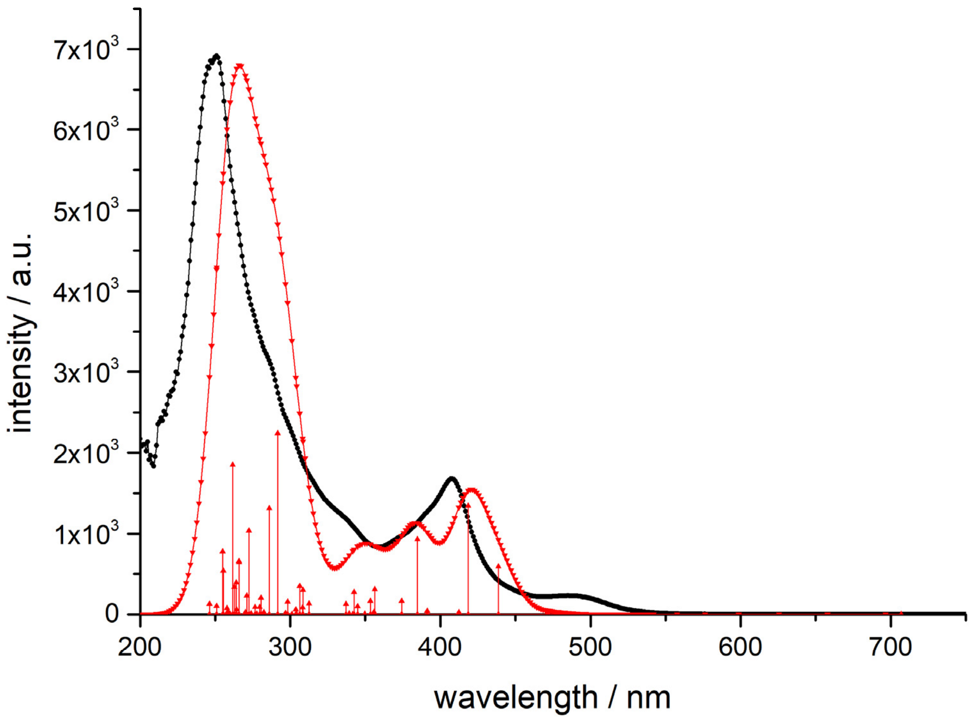


**Figure S6.** Comparison of the measured (black) and the calculated (red) absorption spectrum of complex **9** (calculated with TD-DFT B3LYP/def2-TZVP, CPCM (thf)).

**Table S4.** Calculated states of the absorption spectrum of complex **9** via transition electric dipole moments (TD-DFT: B3LYP/def2-TZVP, RIJCOSX, CPCM (thf)).

| State | Energy | Wavelength | $f_{osc}$ | $T^2$ | $T_X$ | $T_Y$ | $T_Z$ |
|---|---|---|---|---|---|---|---|
| | (cm$^{-1}$) | (nm) | | (a.u.$^2$) | (a.u.) | (a.u.) | (a.u.) |
| **1** | 21213.8 | 471.4 | 0.004630023 | 0.07185 | 0.00986 | -0.24939 | 0.09777 |
| **2** | 22797.2 | 438.7 | 0.082934879 | 1.19765 | -0.01073 | 0.36573 | 1.03140 |
| **3** | 23889.0 | 418.6 | 0.188579920 | 2.59881 | 0.99908 | 1.19196 | -0.42410 |
| **4** | 24251.1 | 412.4 | 0.003409883 | 0.04629 | 0.01337 | 0.08950 | 0.19519 |
| **5** | 25991.3 | 384.7 | 0.131015360 | 1.65947 | 0.79075 | 0.96223 | -0.32910 |
| **6** | 25561.2 | 391.2 | 0.005532032 | 0.07125 | 0.00643 | -0.08755 | -0.25208 |
| **7** | 28301.2 | 353.3 | 0.022701202 | 0.26407 | -0.00478 | 0.17315 | 0.48381 |
| **8** | 28593.4 | 349.7 | 0.000496688 | 0.00572 | 0.07296 | -0.01820 | 0.00798 |
| **9** | 29271.0 | 341.6 | 0.000001764 | 0.00002 | -0.00400 | -0.00066 | -0.00185 |
| **10** | 28064.4 | 356.3 | 0.043515293 | 0.51046 | -0.00456 | -0.24249 | -0.67204 |
| **11** | 26724.0 | 374.2 | 0.022901103 | 0.28212 | 0.40676 | 0.32252 | -0.11243 |
| **12** | 29657.0 | 337.2 | 0.017778180 | 0.19735 | 0.00581 | 0.15580 | 0.41598 |
| **13** | 28105.8 | 355.8 | 0.003733698 | 0.04373 | -0.09517 | -0.17738 | 0.05668 |
| **14** | 29000.3 | 344.8 | 0.013426521 | 0.15242 | 0.28060 | 0.25255 | -0.09950 |
| **15** | 29196.6 | 342.5 | 0.038134435 | 0.42999 | -0.60602 | -0.23562 | 0.08496 |
| **16** | 29474.4 | 339.3 | 0.001437383 | 0.01605 | 0.00210 | -0.04101 | -0.11987 |
| **17** | 31988.5 | 312.6 | 0.018937376 | 0.19490 | 0.00453 | 0.15525 | 0.41324 |
| **18** | 32414.5 | 308.5 | 0.041888976 | 0.42544 | 0.00444 | -0.21768 | -0.61485 |

| | | | | | | | |
|---|---|---|---|---|---|---|---|
| **19** | 32931.4 | 303.7 | 0.008021513 | 0.08019 | -0.01062 | 0.08487 | 0.26995 |
| **20** | 32650.8 | 306.3 | 0.048762361 | 0.49166 | -0.39031 | -0.55207 | 0.18583 |
| **21** | 33220.2 | 301.0 | 0.000206102 | 0.00204 | -0.00120 | 0.01548 | 0.04244 |
| **22** | 32866.4 | 304.3 | 0.000720196 | 0.00721 | -0.00146 | -0.03025 | -0.07935 |
| **23** | 34299.0 | 291.6 | 0.315304368 | 3.02639 | -0.02337 | 0.57250 | 1.64258 |
| **24** | 33529.5 | 298.2 | 0.021790577 | 0.21395 | 0.31381 | 0.33302 | -0.06758 |
| **25** | 32440.2 | 308.3 | 0.011857876 | 0.12034 | -0.21346 | -0.25097 | 0.10855 |
| **26** | 33691.1 | 296.8 | 0.001954004 | 0.01909 | 0.00321 | -0.04770 | -0.12965 |
| **27** | 35420.1 | 282.3 | 0.003113313 | 0.02894 | -0.08083 | -0.14599 | 0.03302 |
| **28** | 35659.1 | 280.4 | 0.028639201 | 0.26440 | -0.28469 | -0.40521 | 0.13843 |
| **29** | 34962.0 | 286.0 | 0.184232511 | 1.73478 | -0.77518 | -1.00729 | 0.34533 |
| **30** | 36149.4 | 276.6 | 0.012427340 | 0.11318 | -0.08066 | 0.30321 | -0.12137 |
| **31** | 36002.7 | 277.8 | 0.000577434 | 0.00528 | 0.01670 | 0.05968 | 0.03794 |
| **32** | 36514.0 | 273.9 | 0.001184153 | 0.01068 | 0.09945 | 0.02803 | 0.00091 |
| **33** | 36699.1 | 272.5 | 0.145629860 | 1.30638 | 0.79800 | 0.77292 | -0.26866 |
| **34** | 37021.4 | 270.1 | 0.003345927 | 0.02975 | 0.02915 | -0.03189 | -0.16699 |
| **35** | 37607.8 | 265.9 | 0.089878066 | 0.78678 | 0.48438 | 0.71451 | -0.20403 |
| **36** | 37842.7 | 264.3 | 0.007903628 | 0.06876 | 0.25381 | 0.06552 | -0.00672 |
| **37** | 37882.1 | 264.0 | 0.055193691 | 0.47966 | 0.01456 | -0.23263 | -0.65217 |
| **38** | 37625.6 | 265.8 | 0.092121367 | 0.80603 | -0.01606 | 0.28969 | 0.84962 |
| **39** | 38061.7 | 262.7 | 0.047036245 | 0.40684 | -0.60046 | -0.20764 | 0.05634 |
| **40** | 35753.9 | 279.7 | 0.012618661 | 0.11619 | 0.12368 | 0.29787 | -0.11029 |
| **41** | 38221.6 | 261.6 | 0.259299310 | 2.23341 | 0.61663 | 1.27412 | -0.47936 |
| **42** | 38771.9 | 257.9 | 0.010479685 | 0.08898 | -0.01211 | 0.09475 | 0.28259 |
| **43** | 38690.2 | 258.5 | 0.004783034 | 0.04070 | 0.18489 | 0.06877 | -0.04226 |
| **44** | 36903.1 | 271.0 | 0.032211880 | 0.28736 | 0.24905 | 0.44360 | -0.16898 |
| **45** | 38477.4 | 259.9 | 0.000256617 | 0.00220 | 0.00672 | 0.02661 | 0.03798 |
| **46** | 39152.5 | 255.4 | 0.075414265 | 0.63412 | 0.71179 | 0.33652 | -0.11931 |
| **47** | 39838.6 | 251.0 | 0.014409692 | 0.11908 | -0.21419 | -0.24221 | 0.12055 |
| **48** | 39852.9 | 250.9 | 0.014541422 | 0.12012 | -0.21616 | -0.26453 | 0.05850 |
| **49** | 39227.5 | 254.9 | 0.108636588 | 0.91172 | 0.00789 | -0.31939 | -0.89980 |
| **50** | 40628.8 | 246.1 | 0.018292183 | 0.14822 | 0.23461 | 0.27100 | -0.14048 |

**Table S5.** Orbital contribution to the first 10 excited states (singlets; HOMO = 218; LUMO = 219) in complex **9**.

| State | The weight of the individual excitations (printed if larger than $10^{-02}$) |
|---|---|
| State 1 | 218a -> 219a : 0.960932 (c= -0.98027151) |
| State 2 | 214a -> 219a : 0.011465 (c= 0.10707332)<br>218a -> 220a : 0.951401 (c= 0.97539781) |
| State 3 | 217a -> 219a : 0.967014 (c= -0.98336847) |
| State 4 | 217a -> 220a : 0.945919 (c= -0.97258347)<br>218a -> 222a : 0.029286 (c= 0.17113152) |
| State 5 | 218a -> 221a : 0.948712 (c= 0.97401834) |
| State 6 | 212a -> 219a : 0.012664 (c= -0.11253371)<br>214a -> 219a : 0.020422 (c= 0.14290478)<br>215a -> 219a : 0.028540 (c= -0.16893831)<br>216a -> 219a : 0.854188 (c= -0.92422282) |

| | |
|---|---|
| | 218a -> 222a : 0.047831 (c= -0.21870189) |
| State 7 | 211a -> 219a : 0.018359 (c= 0.13549677)<br>212a -> 219a : 0.017196 (c= 0.13113338)<br>214a -> 219a : 0.048981 (c= 0.22131541)<br>215a -> 219a : 0.639701 (c= -0.79981323)<br>216a -> 219a : 0.059836 (c= 0.24461361)<br>218a -> 222a : 0.157704 (c= -0.39711950)<br>218a -> 223a : 0.021042 (c= -0.14505918) |
| State 8 | 215a -> 220a : 0.017268 (c= -0.13140617)<br>216a -> 220a : 0.029307 (c= 0.17119418)<br>217a -> 221a : 0.893987 (c= -0.94550890) |
| State 9 | 211a -> 219a : 0.012899 (c= -0.11357334)<br>215a -> 219a : 0.198269 (c= 0.44527416)<br>216a -> 219a : 0.015998 (c= 0.12648243)<br>216a -> 221a : 0.011684 (c= 0.10809412)<br>217a -> 220a : 0.024984 (c= -0.15806215)<br>218a -> 222a : 0.687201 (c= -0.82897565) |
| State 10 | 212a -> 219a : 0.036845 (c= 0.19195052)<br>214a -> 219a : 0.806934 (c= 0.89829499)<br>215a -> 219a : 0.028787 (c= 0.16966845)<br>218a -> 222a : 0.026087 (c= 0.16151559)<br>218a -> 223a : 0.046455 (c= 0.21553373) |

## b) *Calculated UV/Vis spectra of **1** and **7**.*

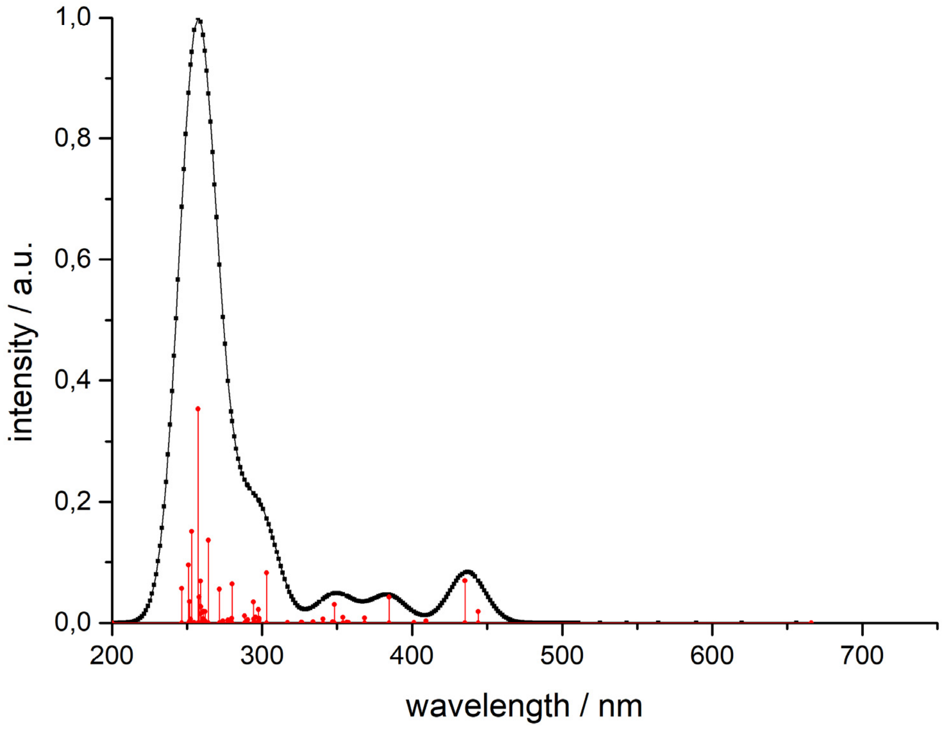


**Figure S7.** Calculated absorption spectrum of **1** (calculated with TD-DFT B3LYP/def2-TZVP, CPCM (thf)).

**Table S6.** Calculated states of the absorption spectrum of **1** via transition electric dipole moments (TD-DFT: B3LYP/def2-TZVP, RIJCOSX, CPCM (thf)).

| State | Energy | Wavelength | $f_{osc}$ | $T^2$ | $T_X$ | $T_Y$ | $T_Z$ |
|---|---|---|---|---|---|---|---|
| | ($cm^{-1}$) | (nm) | | (a.u.$^2$) | (a.u.) | (a.u.) | (a.u.) |
| **1** | 22520.5 | 444.0 | 0.021463756 | 0.31376 | -0.09637 | -0.50962 | 0.21157 |
| **2** | 22977.5 | 435.2 | 0.081290261 | 1.16469 | 0.25381 | 0.99941 | 0.31852 |
| **3** | 24426.3 | 409.4 | 0.003600337 | 0.04852 | 0.21718 | -0.03423 | -0.01356 |
| **4** | 24920.1 | 401.3 | 0.000748358 | 0.00989 | -0.08795 | 0.04255 | 0.01846 |
| **5** | 25999.2 | 384.6 | 0.049679517 | 0.62906 | -0.17895 | -0.74072 | -0.21994 |
| **6** | 27148.2 | 368.3 | 0.009621195 | 0.11667 | 0.07595 | 0.22028 | 0.24976 |
| **7** | 28245.8 | 354.0 | 0.011001089 | 0.12822 | 0.34877 | -0.07516 | -0.03049 |
| **8** | 28707.1 | 348.3 | 0.035344573 | 0.40533 | 0.61814 | -0.14118 | -0.05750 |
| **9** | 29937.6 | 334.0 | 0.001598736 | 0.01758 | 0.03127 | 0.10671 | 0.07223 |
| **10** | 28048.0 | 356.5 | 0.001168578 | 0.01372 | -0.11693 | -0.00424 | 0.00499 |
| **11** | 27964.5 | 357.6 | 0.000652178 | 0.00768 | 0.02289 | -0.08088 | -0.02473 |
| **12** | 30600.7 | 326.8 | 0.000565748 | 0.00609 | 0.02657 | 0.06870 | -0.02570 |
| **13** | 30650.0 | 326.3 | 0.001273871 | 0.01368 | -0.11319 | 0.02793 | 0.00952 |
| **14** | 31556.1 | 316.9 | 0.000220871 | 0.00230 | -0.04135 | 0.02192 | 0.01066 |
| **15** | 28823.0 | 346.9 | 0.001564054 | 0.01786 | -0.03271 | -0.12099 | -0.04643 |
| **16** | 29364.0 | 340.6 | 0.007586449 | 0.08505 | -0.07232 | -0.26217 | -0.10531 |
| **17** | 33613.2 | 297.5 | 0.026100159 | 0.25563 | -0.49344 | 0.10173 | 0.04235 |
| **18** | 34439.2 | 290.4 | 0.006303647 | 0.06026 | -0.23783 | 0.05550 | 0.02479 |

| | | | | | | | |
|---|---|---|---|---|---|---|---|
| **19** | 34686.5 | 288.3 | 0.013741678 | 0.13042 | 0.04838 | 0.35095 | 0.07011 |
| **20** | 33965.3 | 294.4 | 0.007642454 | 0.07408 | -0.26599 | -0.05733 | -0.00602 |
| **21** | 33818.0 | 295.7 | 0.011356403 | 0.11055 | 0.01856 | 0.31912 | 0.09149 |
| **22** | 33559.4 | 298.0 | 0.008933228 | 0.08763 | 0.28979 | -0.05576 | -0.02335 |
| **23** | 33557.6 | 298.0 | 0.005486470 | 0.05382 | 0.08255 | -0.20935 | -0.05640 |
| **24** | 35701.4 | 280.1 | 0.074883345 | 0.69052 | 0.81588 | -0.14302 | -0.06632 |
| **25** | 33979.7 | 294.3 | 0.040488738 | 0.39227 | -0.60587 | 0.14806 | 0.05722 |
| **26** | 32989.8 | 303.1 | 0.096404903 | 0.96205 | -0.95342 | 0.21177 | 0.09046 |
| **27** | 35747.5 | 279.7 | 0.008782486 | 0.08088 | 0.06515 | 0.25613 | 0.10504 |
| **28** | 36817.6 | 271.6 | 0.064774537 | 0.57919 | -0.74191 | 0.15753 | 0.06283 |
| **29** | 36059.8 | 277.3 | 0.005361414 | 0.04895 | -0.03459 | -0.10456 | -0.19188 |
| **30** | 37837.3 | 264.3 | 0.159729042 | 1.38976 | 1.14275 | -0.26665 | -0.11301 |
| **31** | 38328.3 | 260.9 | 0.022094328 | 0.18977 | -0.14818 | -0.40231 | -0.07720 |
| **32** | 38187.8 | 261.9 | 0.004903693 | 0.04227 | 0.19095 | -0.07140 | -0.02675 |
| **33** | 35797.9 | 279.3 | 0.004372049 | 0.04021 | 0.06594 | 0.10753 | -0.15587 |
| **34** | 38606.7 | 259.0 | 0.080612708 | 0.68741 | -0.81051 | 0.15540 | 0.07957 |
| **35** | 38618.0 | 258.9 | 0.017693129 | 0.15083 | -0.28422 | 0.26464 | -0.00401 |
| **36** | 38140.4 | 262.2 | 0.021501607 | 0.18559 | 0.39034 | -0.18045 | -0.02578 |
| **37** | 38865.7 | 257.3 | 0.413264034 | 3.50056 | -1.82948 | 0.35225 | 0.17167 |
| **38** | 38748.4 | 258.1 | 0.049593615 | 0.42135 | -0.13401 | -0.61348 | -0.16443 |
| **39** | 38008.6 | 263.1 | 0.000572069 | 0.00495 | -0.06644 | 0.02159 | 0.00862 |
| **40** | 38569.3 | 259.3 | 0.031584344 | 0.26959 | 0.12594 | 0.50370 | 0.00388 |
| **41** | 36506.3 | 273.9 | 0.003657240 | 0.03298 | 0.13713 | -0.10887 | -0.04820 |
| **42** | 39733.6 | 251.7 | 0.041236866 | 0.34167 | 0.17930 | 0.51220 | 0.21720 |
| **43** | 39514.2 | 253.1 | 0.177268395 | 1.47691 | -0.25398 | -1.18414 | 0.10112 |
| **44** | 39636.4 | 252.3 | 0.007621371 | 0.06330 | 0.12707 | 0.20442 | 0.07325 |
| **45** | 39697.2 | 251.9 | 0.003908774 | 0.03242 | -0.06960 | 0.13415 | 0.09786 |
| **46** | 39243.9 | 254.8 | 0.000620941 | 0.00521 | -0.01275 | -0.04489 | 0.05506 |
| **47** | 38372.1 | 260.6 | 0.007769103 | 0.06665 | 0.00456 | 0.25442 | -0.04365 |
| **48** | 34494.1 | 289.9 | 0.003632616 | 0.03467 | 0.00730 | -0.18519 | 0.01788 |
| **49** | 40575.4 | 246.5 | 0.066556408 | 0.54001 | 0.72193 | -0.11847 | -0.06924 |
| **50** | 39860.7 | 250.9 | 0.111607597 | 0.92177 | -0.92818 | 0.22837 | 0.09004 |

**Table S7.** Orbital contribution to the first 10 excited states (singlets; HOMO = 191; LUMO = 192) in **1**.

| State | The weight of the individual excitations (printed if larger than $10^{-02}$) |
|---|---|
| State 1 | 190a -> 192a : 0.010703 (c= 0.10345527)<br>191a -> 192a : 0.956871 (c= 0.97819758) |
| State 2 | 190a -> 192a : 0.950483 (c= 0.97492713)<br>191a -> 192a : 0.010905 (c= -0.10442616)<br>191a -> 193a : 0.011527 (c= -0.10736397) |
| State 3 | 190a -> 194a : 0.027868 (c= -0.16693576)<br>191a -> 194a : 0.958181 (c= -0.97886696) |
| State 4 | 190a -> 194a : 0.953767 (c= -0.97660975)<br>191a -> 194a : 0.028324 (c= 0.16829848) |
| State 5 | 190a -> 192a : 0.012805 (c= 0.11315775)<br>190a -> 195a : 0.010193 (c= -0.10095912) |

| | |
|---|---|
| | 191a -> 193a : 0.941346 (c= 0.97023001) |
| State 6 | 190a -> 193a : 0.926029 (c= -0.96230390)<br>191a -> 195a : 0.021208 (c= 0.14563006) |
| State 7 | 186a -> 194a : 0.010592 (c= 0.10291972)<br>188a -> 192a : 0.036733 (c= 0.19165956)<br>189a -> 192a : 0.811913 (c= 0.90106225)<br>190a -> 197a : 0.014698 (c= 0.12123417)<br>191a -> 196a : 0.052220 (c= 0.22851728)<br>191a -> 197a : 0.022569 (c= -0.15022995) |
| State 8 | 188a -> 192a : 0.819504 (c= -0.90526472)<br>189a -> 192a : 0.028815 (c= 0.16975039)<br>190a -> 196a : 0.065553 (c= -0.25603390)<br>190a -> 197a : 0.016654 (c= -0.12905196)<br>191a -> 197a : 0.039952 (c= -0.19987908) |
| State 9 | 186a -> 192a : 0.062514 (c= -0.25002762)<br>187a -> 194a : 0.029783 (c= -0.17257682)<br>190a -> 193a : 0.027926 (c= -0.16710942)<br>190a -> 195a : 0.017246 (c= -0.13132380)<br>191a -> 195a : 0.823812 (c= -0.90764103) |
| State 10 | 186a -> 192a : 0.092633 (c= 0.30435660)<br>186a -> 194a : 0.192544 (c= 0.43879788)<br>187a -> 192a : 0.543614 (c= 0.73730195)<br>187a -> 194a : 0.038572 (c= 0.19639638)<br>187a -> 195a : 0.028456 (c= -0.16869026)<br>189a -> 192a : 0.012660 (c= -0.11251616)<br>191a -> 195a : 0.037067 (c= -0.19252733) |

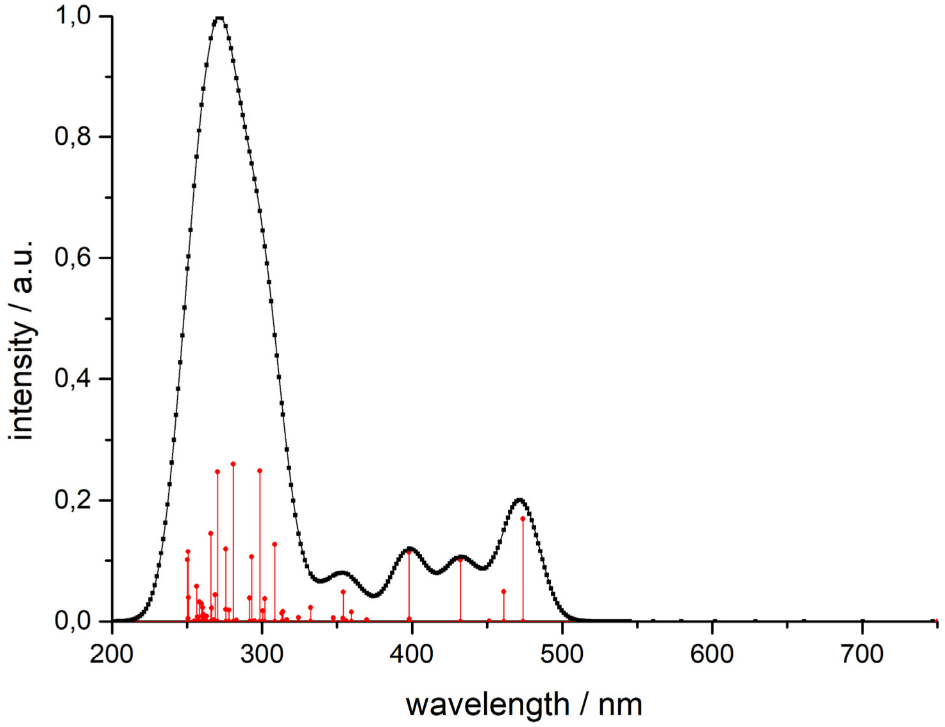


**Figure S8.** Calculated absorption spectrum of complex **7** (calculated with TD-DFT B3LYP/def2-TZVP, CPCM (thf)).

**Table S8.** Calculated states of the absorption spectrum of complex **7** via transition electric dipole moments (TD-DFT: B3LYP/def2-TZVP, RIJCOSX, CPCM (thf)).

| State | Energy | Wavelength | $f_{osc}$ | $T^2$ | $T_X$ | $T_Y$ | $T_Z$ |
|---|---|---|---|---|---|---|---|
| | ($cm^{-1}$) | (nm) | | (a.u.$^2$) | (a.u.) | (a.u.) | (a.u.) |
| 1 | 21100.2 | 473.9 | 0.125229874 | 1.95387 | -0.87524 | -1.03072 | 0.35416 |
| 2 | 21687.1 | 461.1 | 0.036046633 | 0.54719 | 0.46305 | 0.54495 | -0.18924 |
| 3 | 22161.1 | 451.2 | 0.000131382 | 0.00195 | -0.02475 | 0.00238 | -0.03651 |
| 4 | 23133.9 | 432.3 | 0.074344664 | 1.05798 | 0.16693 | -0.46353 | -0.90291 |
| 5 | 25123.8 | 398.0 | 0.084242364 | 1.10388 | -0.66132 | -0.76994 | 0.27153 |
| 6 | 25111.9 | 398.2 | 0.002709402 | 0.03552 | -0.11882 | -0.13683 | 0.05175 |
| 7 | 27051.3 | 369.7 | 0.002116646 | 0.02576 | 0.00496 | -0.05451 | -0.15088 |
| 8 | 28092.1 | 356.0 | 0.000913813 | 0.01071 | 0.06364 | 0.07723 | -0.02635 |
| 9 | 27804.9 | 359.6 | 0.011620377 | 0.13759 | -0.01838 | 0.13891 | 0.34344 |
| 10 | 28236.2 | 354.2 | 0.035756346 | 0.41689 | -0.02499 | 0.22969 | 0.60292 |
| 11 | 30093.1 | 332.3 | 0.016574106 | 0.18132 | -0.00264 | -0.14385 | -0.40077 |
| 12 | 28253.1 | 353.9 | 0.003566984 | 0.04156 | 0.12901 | 0.14450 | -0.06357 |
| 13 | 28775.7 | 347.5 | 0.004210318 | 0.04817 | 0.13772 | 0.15970 | -0.06080 |
| 14 | 30836.2 | 324.3 | 0.004483385 | 0.04787 | 0.00575 | -0.07714 | -0.20465 |
| 15 | 31932.3 | 313.2 | 0.009669206 | 0.09969 | 0.19838 | 0.23054 | -0.08474 |
| 16 | 31599.8 | 316.5 | 0.002068387 | 0.02155 | 0.09089 | -0.05991 | 0.09848 |
| 17 | 32408.2 | 308.6 | 0.093797739 | 0.95282 | -0.12827 | 0.38871 | 0.88616 |
| 18 | 31852.5 | 313.9 | 0.012173697 | 0.12582 | -0.21138 | -0.28188 | 0.04103 |

| | | | | | | | |
|---|---|---|---|---|---|---|---|
| 19 | 33276.5 | 300.5 | 0.012892679 | 0.12755 | 0.18072 | -0.23136 | -0.20337 |
| 20 | 33490.0 | 298.6 | 0.183338516 | 1.80225 | 0.02830 | 0.43308 | 1.27039 |
| 21 | 33117.8 | 302.0 | 0.027721549 | 0.27557 | 0.32910 | 0.37887 | -0.15402 |
| 22 | 33893.6 | 295.0 | 0.000811485 | 0.00788 | -0.01641 | 0.03334 | 0.08063 |
| 23 | 34284.5 | 291.7 | 0.028356920 | 0.27229 | 0.11689 | -0.21356 | -0.46154 |
| 24 | 34114.4 | 293.1 | 0.078245351 | 0.75509 | 0.53999 | 0.65069 | -0.20024 |
| 25 | 35351.1 | 282.9 | 0.001766800 | 0.01645 | -0.01201 | 0.05531 | 0.11511 |
| 26 | 35993.2 | 277.8 | 0.013832874 | 0.12652 | -0.02900 | -0.08622 | -0.34387 |
| 27 | 36270.9 | 275.7 | 0.014469548 | 0.13133 | -0.22483 | -0.27107 | 0.08548 |
| 28 | 35609.6 | 280.8 | 0.191508617 | 1.77050 | -0.83259 | -0.98192 | 0.33633 |
| 29 | 36248.1 | 275.9 | 0.088282608 | 0.80180 | 0.04398 | -0.31991 | -0.83518 |
| 30 | 36991.7 | 270.3 | 0.182061472 | 1.62028 | -0.00803 | 0.42704 | 1.19910 |
| 31 | 37210.2 | 268.7 | 0.032301035 | 0.28578 | 0.03471 | -0.18459 | -0.50050 |
| 32 | 37552.3 | 266.3 | 0.016326520 | 0.14313 | -0.23314 | -0.28210 | 0.09589 |
| 33 | 37627.2 | 265.8 | 0.107316531 | 0.93895 | -0.60456 | -0.71446 | 0.25100 |
| 34 | 37321.3 | 267.9 | 0.002251094 | 0.01986 | -0.08789 | -0.04710 | -0.09957 |
| 35 | 38035.5 | 262.9 | 0.000469599 | 0.00406 | -0.03771 | -0.05034 | 0.01044 |
| 36 | 38407.4 | 260.4 | 0.016902241 | 0.14488 | -0.05219 | 0.15772 | 0.34246 |
| 37 | 38320.7 | 261.0 | 0.002667000 | 0.02291 | -0.09318 | -0.10534 | 0.05596 |
| 38 | 38026.2 | 263.0 | 0.006471820 | 0.05603 | 0.02565 | 0.02618 | 0.23385 |
| 39 | 38450.8 | 260.1 | 0.006378825 | 0.05461 | -0.14511 | -0.17820 | 0.04245 |
| 40 | 38489.2 | 259.8 | 0.021388019 | 0.18294 | -0.26588 | -0.31763 | 0.10657 |
| 41 | 38724.2 | 258.2 | 0.023493508 | 0.19973 | 0.28495 | 0.31570 | -0.13736 |
| 42 | 38993.1 | 256.5 | 0.042733438 | 0.36079 | -0.10872 | 0.30322 | 0.50698 |
| 43 | 38318.0 | 261.0 | 0.008467366 | 0.07275 | -0.17146 | 0.05370 | -0.20116 |
| 44 | 38746.9 | 258.1 | 0.000273104 | 0.00232 | -0.01165 | 0.04283 | 0.01871 |
| 45 | 38931.7 | 256.9 | 0.005485039 | 0.04638 | 0.11894 | -0.12994 | -0.12390 |
| 46 | 39248.8 | 254.8 | 0.000352130 | 0.00295 | 0.02809 | 0.04377 | -0.01577 |
| 47 | 39882.6 | 250.7 | 0.003715958 | 0.03067 | 0.10629 | -0.09128 | 0.10509 |
| 48 | 39899.8 | 250.6 | 0.084752270 | 0.69929 | 0.10452 | -0.09360 | -0.82438 |
| 49 | 39944.7 | 250.3 | 0.074822219 | 0.61666 | -0.42321 | -0.62699 | -0.21081 |
| 50 | 39846.3 | 251.0 | 0.028721602 | 0.23730 | 0.20003 | 0.02926 | -0.44321 |

**Table S9.** Orbital contribution to the first 10 excited states (singlets; HOMO = 273; LUMO = 274) in complex **7**.

| State | The weight of the individual excitations (printed if larger than $10^{-02}$) |
|---|---|
| State 1 | 272a -> 274a : 0.716466 (c= 0.84644305)<br>273a -> 274a : 0.265014 (c= 0.51479541) |
| State 2 | 272a -> 274a : 0.261694 (c= -0.51156068)<br>273a -> 274a : 0.707008 (c= 0.84083757) |
| State 3 | 272a -> 275a : 0.948735 (c= 0.97403051)<br>273a -> 275a : 0.032684 (c= 0.18078813) |
| State 4 | 272a -> 275a : 0.029276 (c= 0.17110196)<br>273a -> 275a : 0.934200 (c= -0.96654003) |
| State 5 | 273a -> 276a : 0.952436 (c= -0.97592829) |
| State 6 | 272a -> 276a : 0.957613 (c= 0.97857691) |

| | |
|---|---|
| State 7 | 266a -> 275a : 0.067519 (c= 0.25984482)<br>268a -> 274a : 0.064257 (c= -0.25348928)<br>269a -> 274a : 0.462138 (c= -0.67980753)<br>270a -> 274a : 0.062745 (c= 0.25049006)<br>271a -> 274a : 0.316034 (c= -0.56216881) |
| State 8 | 266a -> 274a : 0.149798 (c= 0.38703699)<br>266a -> 278a : 0.010517 (c= 0.10255383)<br>268a -> 275a : 0.067171 (c= -0.25917367)<br>269a -> 275a : 0.452935 (c= -0.67300434)<br>270a -> 275a : 0.057871 (c= 0.24056316)<br>271a -> 275a : 0.246196 (c= -0.49618114) |
| State 9 | 269a -> 274a : 0.282615 (c= 0.53161531)<br>270a -> 274a : 0.026776 (c= -0.16363382)<br>271a -> 274a : 0.534493 (c= -0.73109038)<br>272a -> 277a : 0.015638 (c= -0.12505016)<br>273a -> 277a : 0.091594 (c= -0.30264433) |
| State 10 | 267a -> 274a : 0.018424 (c= 0.13573374)<br>269a -> 274a : 0.095908 (c= -0.30969050)<br>270a -> 274a : 0.774440 (c= -0.88002255)<br>272a -> 277a : 0.053363 (c= -0.23100537)<br>272a -> 279a : 0.014952 (c= -0.12227734)<br>273a -> 279a : 0.011683 (c= 0.10808831) |

# 6. NMR Spectra

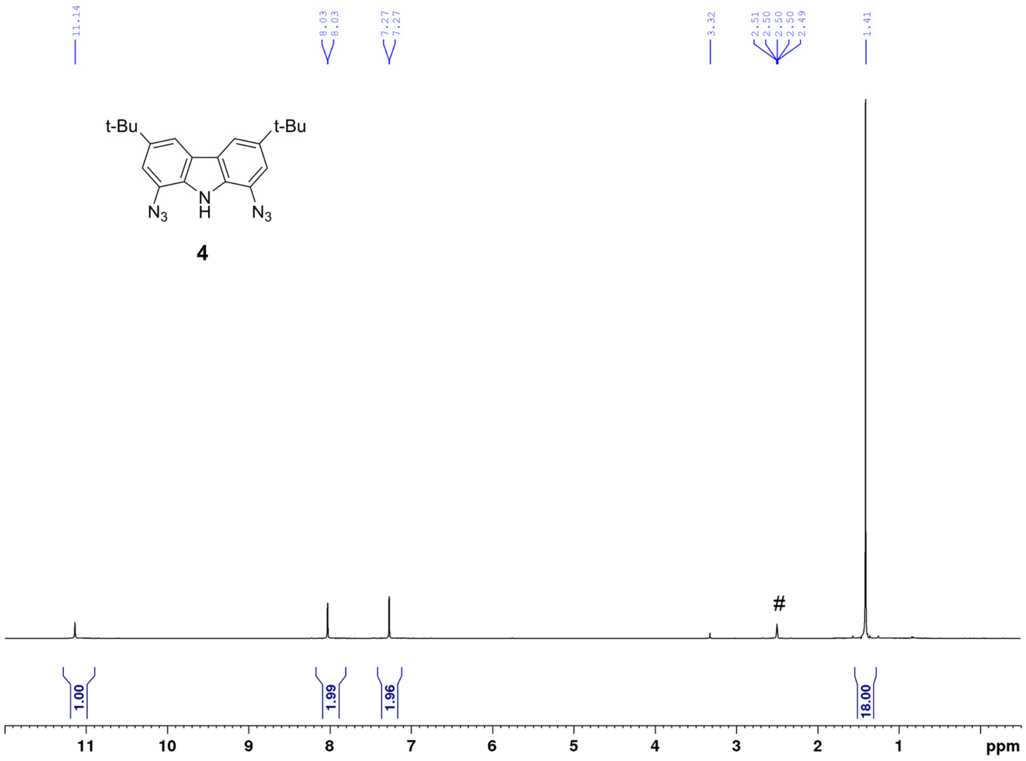


**Figure S9.** $^{1}H$ NMR (DMSO-$d_6$ (#), 400 MHz) spectrum of **4**.

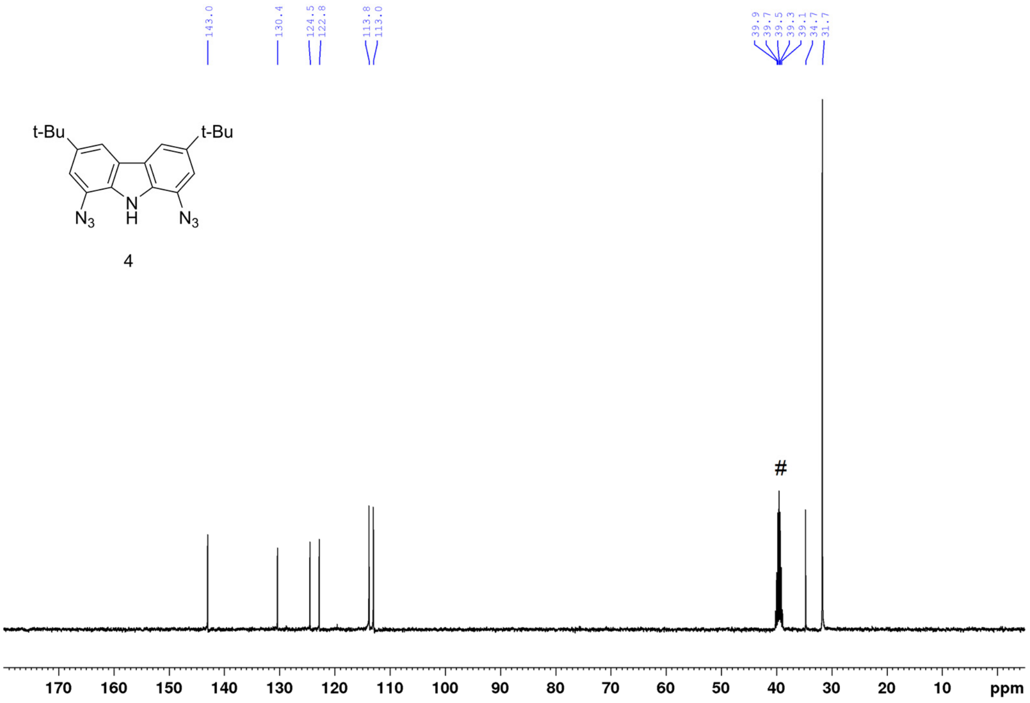


**Figure S10.** $^{13}C$ NMR (DMSO-$d_6$ (#), 100 MHz) spectrum of **4**.

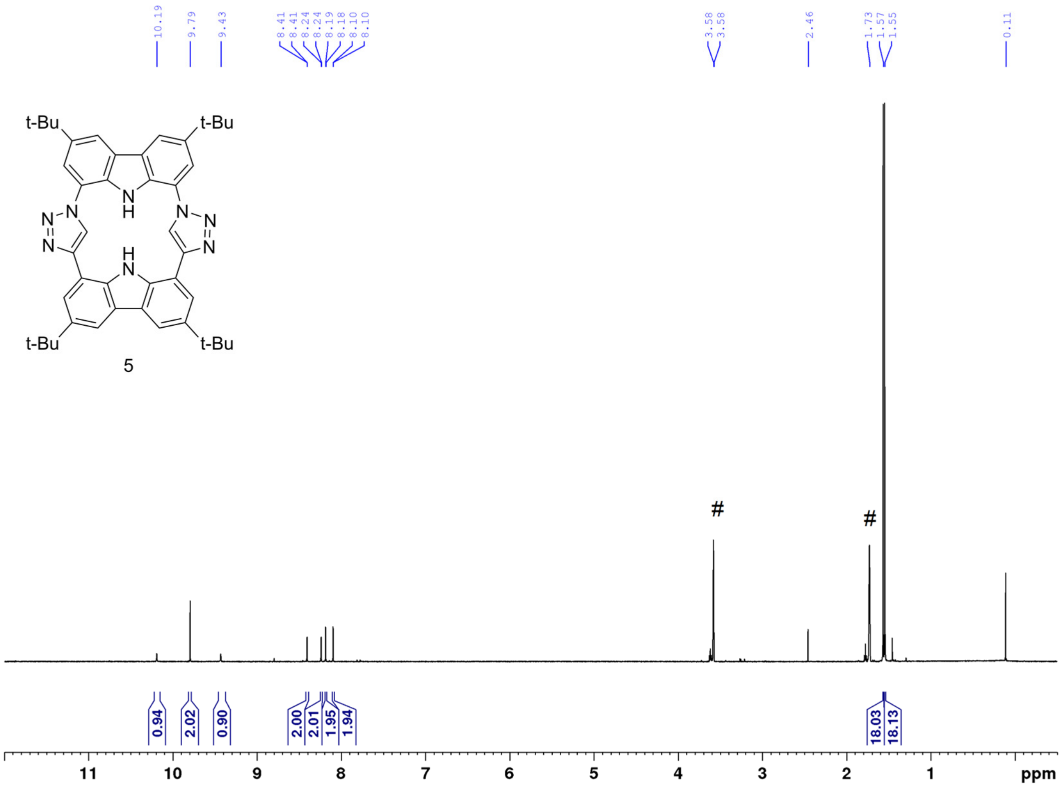


**Figure S11.** $^{1}$H NMR (THF-$d_8$ (#), 500 MHz) spectrum of **5**.

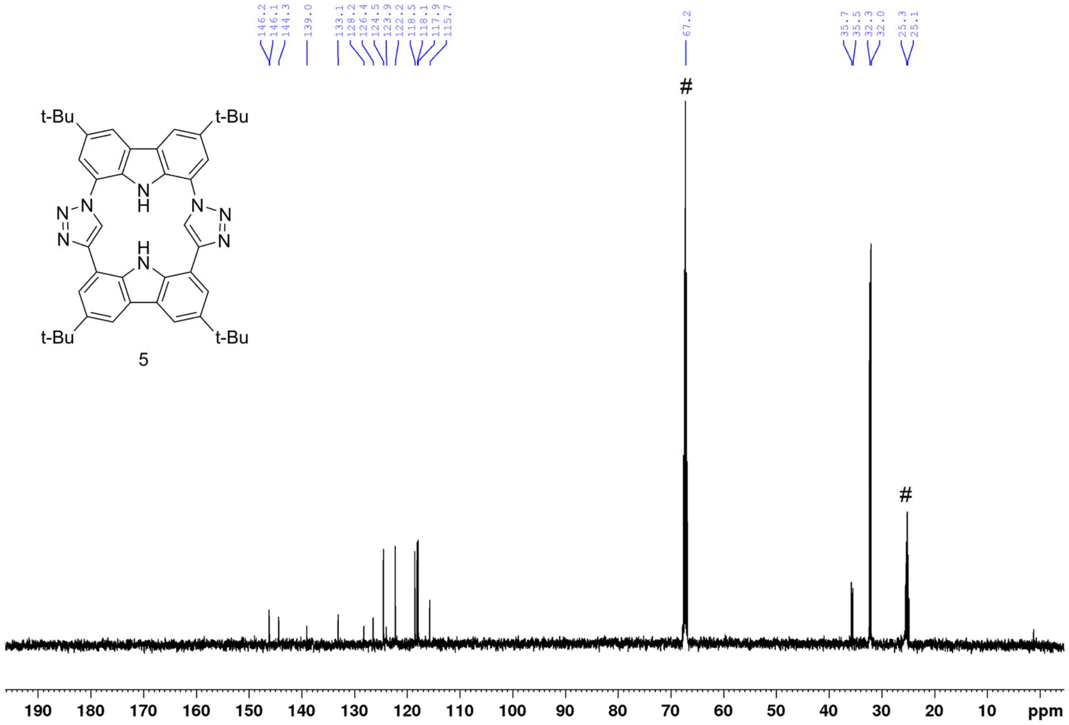


**Figure S12.** $^{13}$C NMR (THF-$d_8$ (#), 125 MHz) spectrum of **5**.

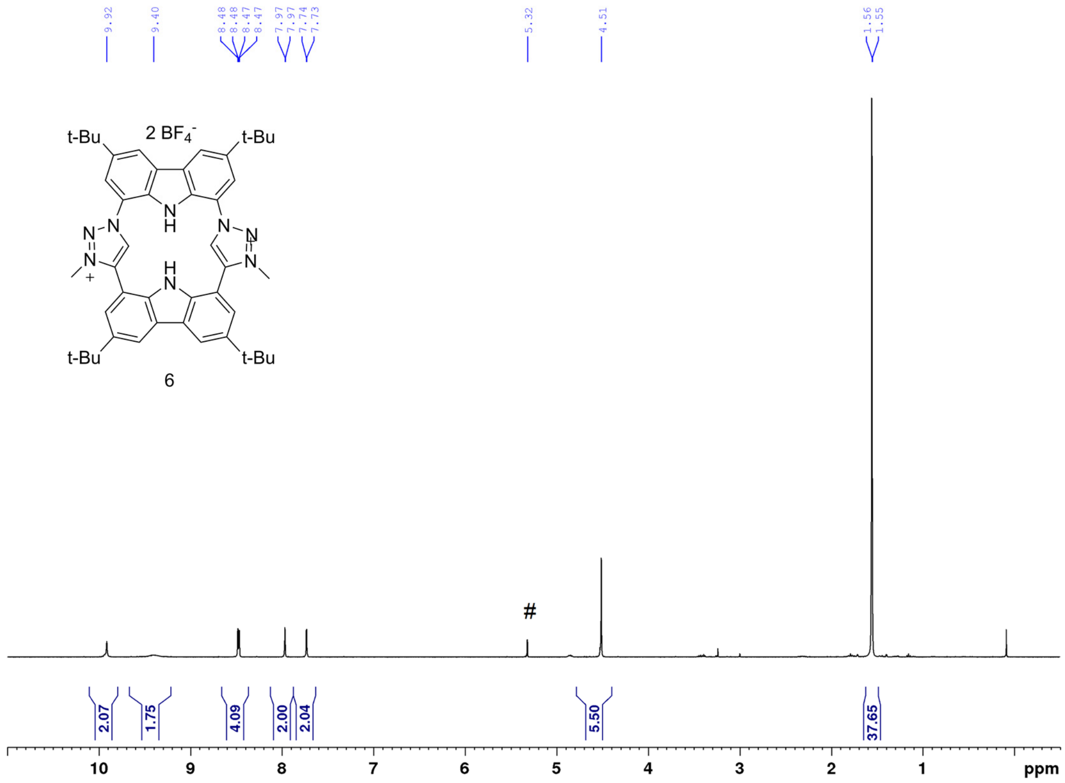


**Figure S13.** $^{1}$H NMR ($CD_2Cl_2$ (#), 400 MHz) spectrum of **6**.

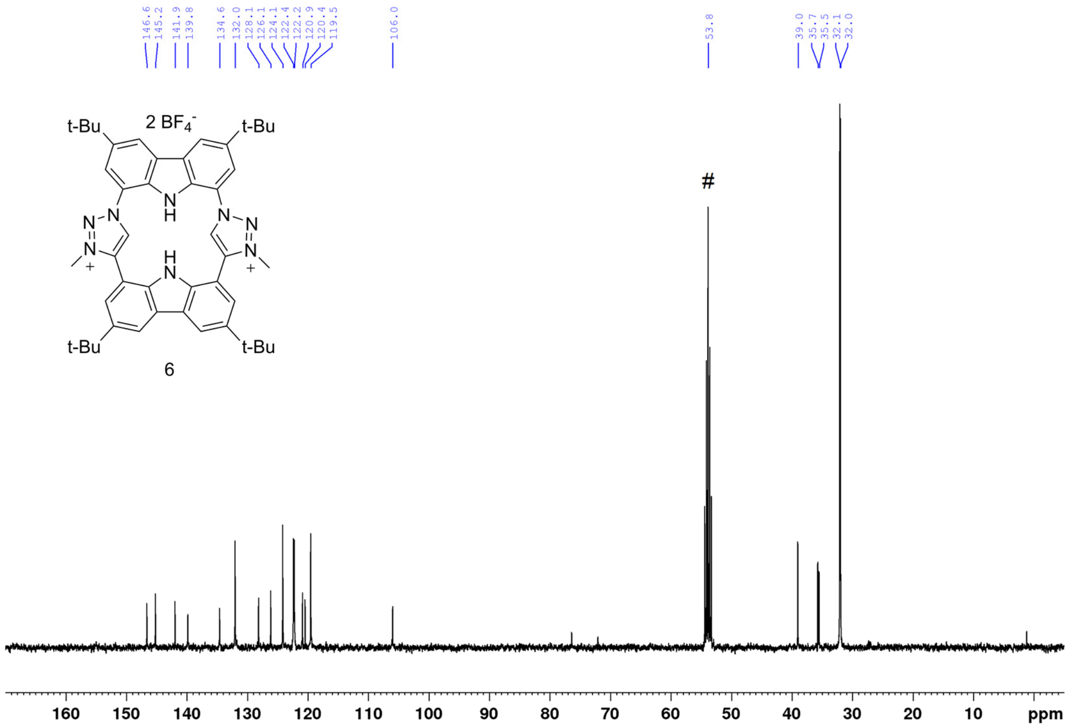


**Figure S14.** $^{13}$C NMR ($CD_2Cl_2$ (#), 100 MHz) spectrum of **6**.

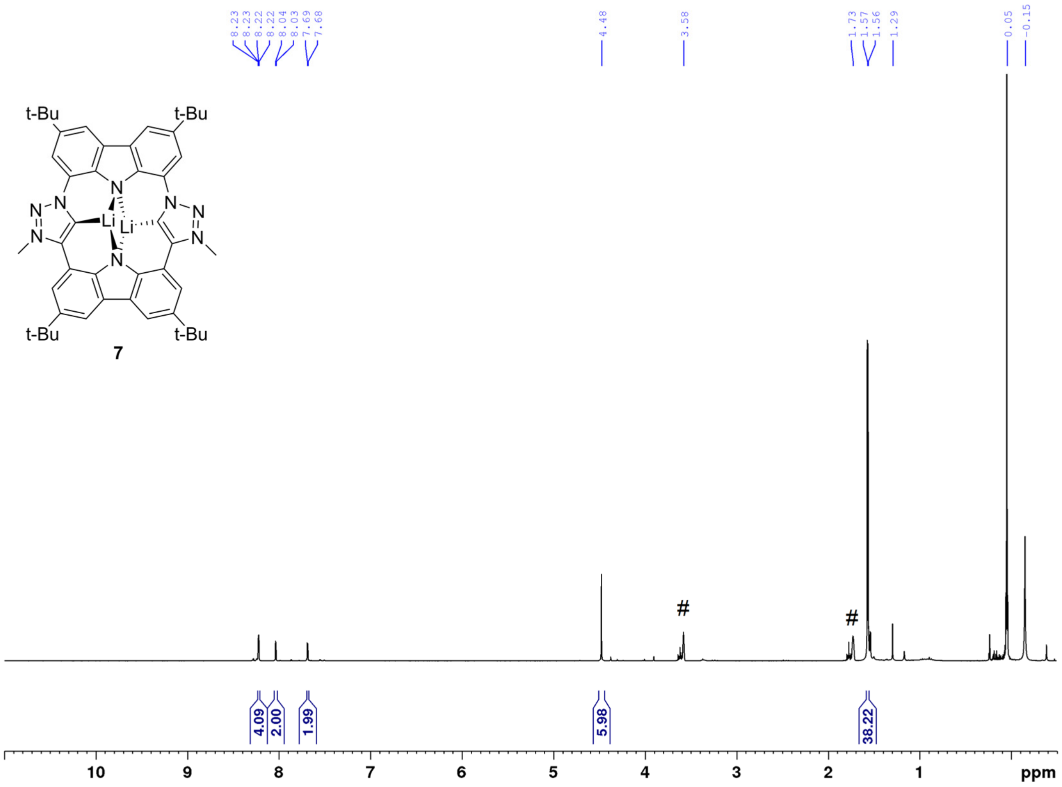


**Figure S15.** $^{1}H$ NMR (THF-$d_8$ (#), 400 MHz) spectrum of **7**.

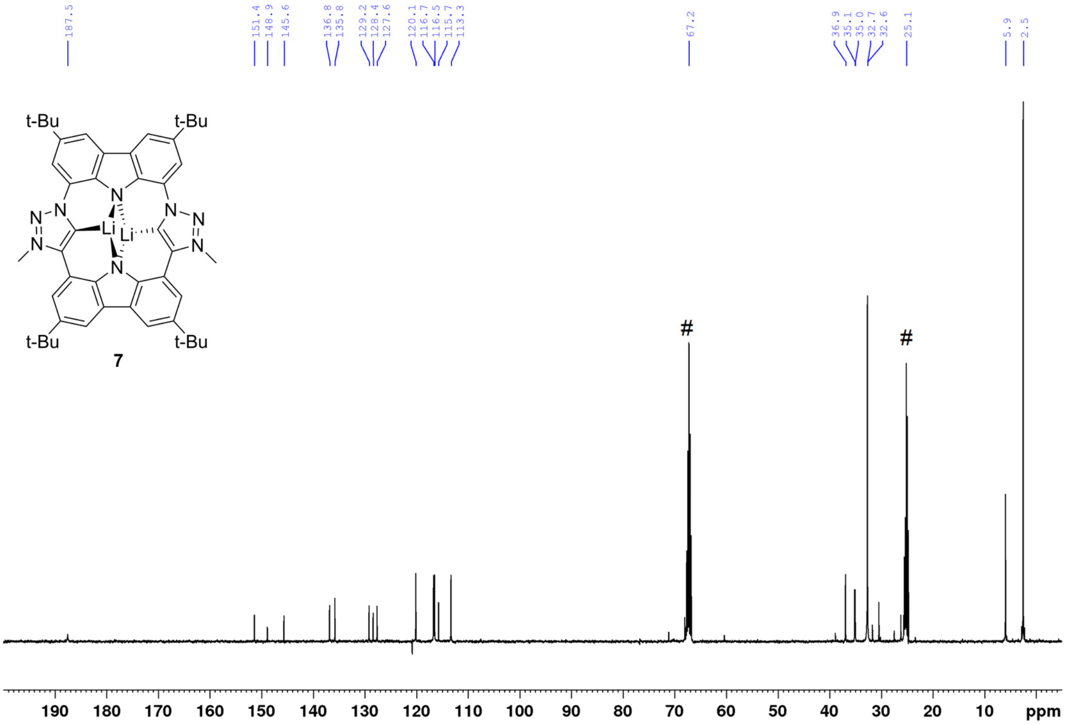


**Figure S16.** $^{13}C$ NMR (THF-$d_8$ (#), 100 MHz) spectrum of **7** at 26 °C.

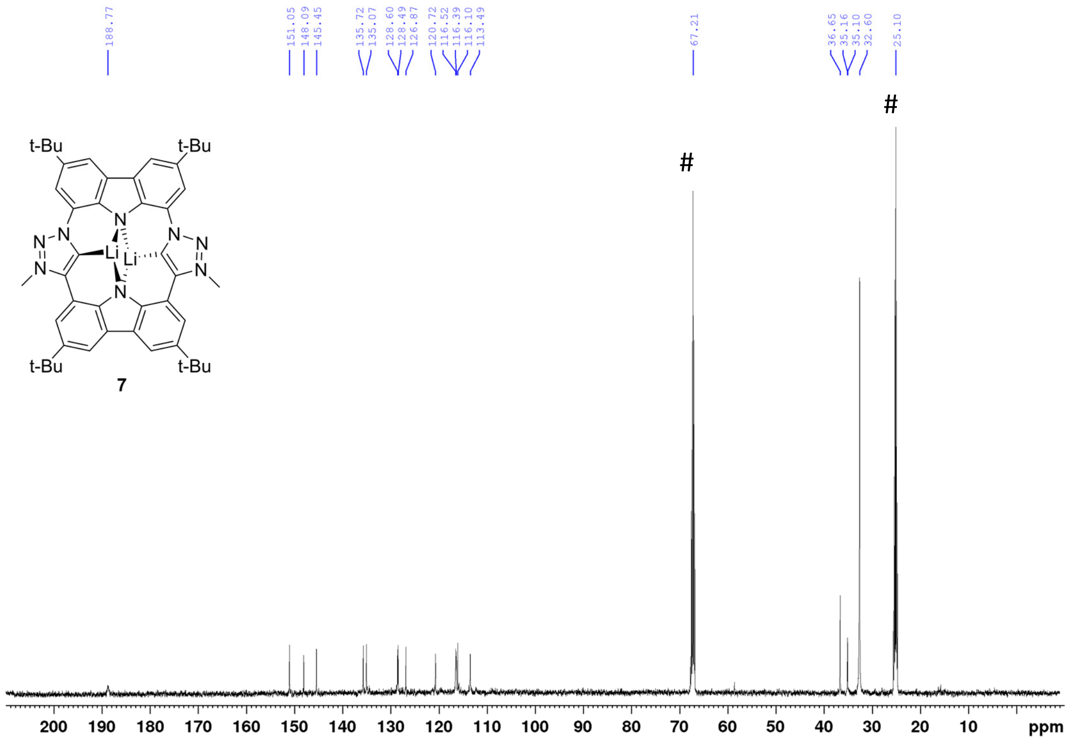


**Figure S17.** $^{13}$C NMR (THF-d$_8$ (#), 125 MHz) spectrum of complex **7** at -80 °C.

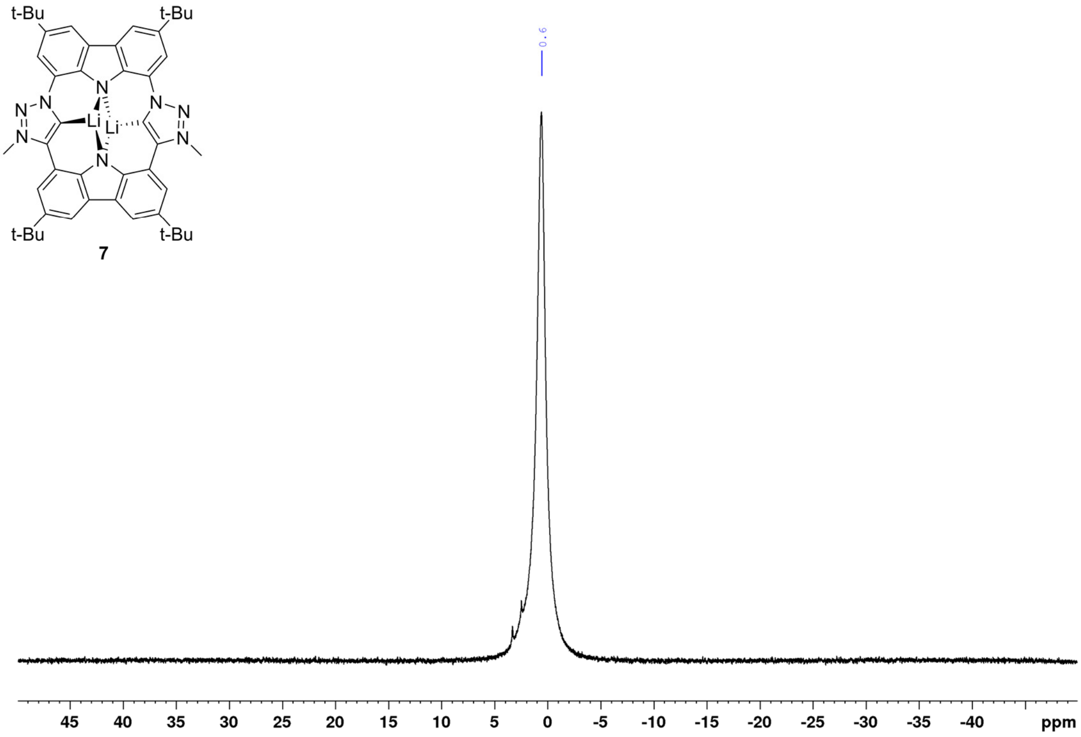


**Figure S18.** $^{7}$Li NMR (THF-d$_8$, 117 MHz) spectrum of **7** at 26 °C.

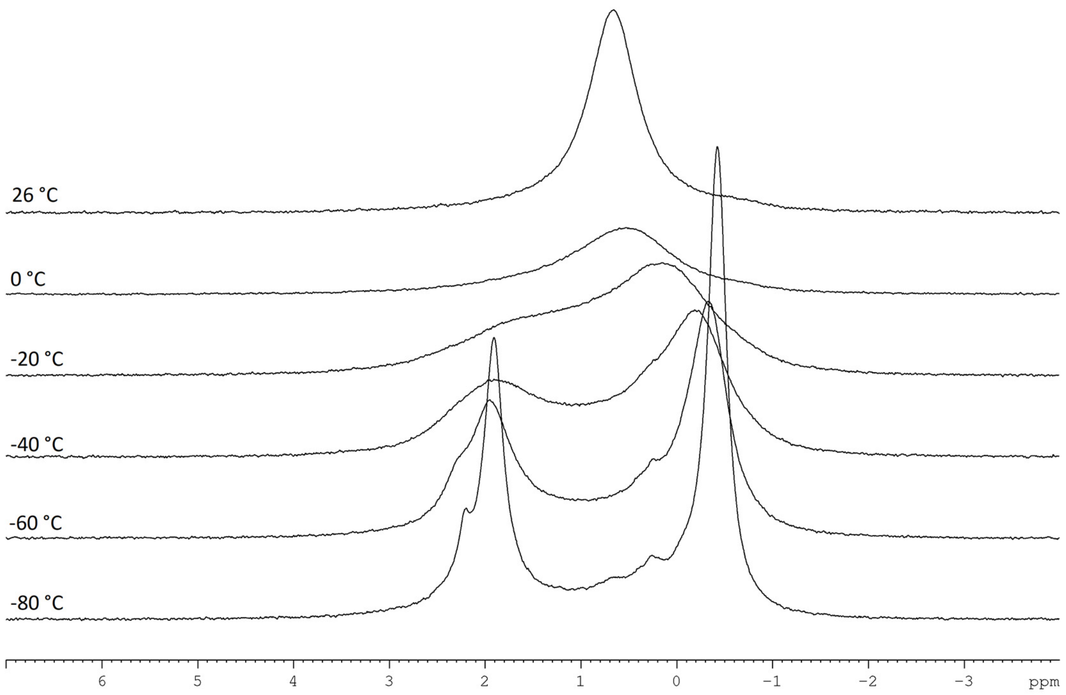


**Figure S19.** $^{7}$Li NMR (THF-d$_8$, 117 MHz) spectra of complex **7** at various temperatures. Peak at 1.90 ppm for the lithium complex **7** and at -0.43 ppm for $LiBF_4$.

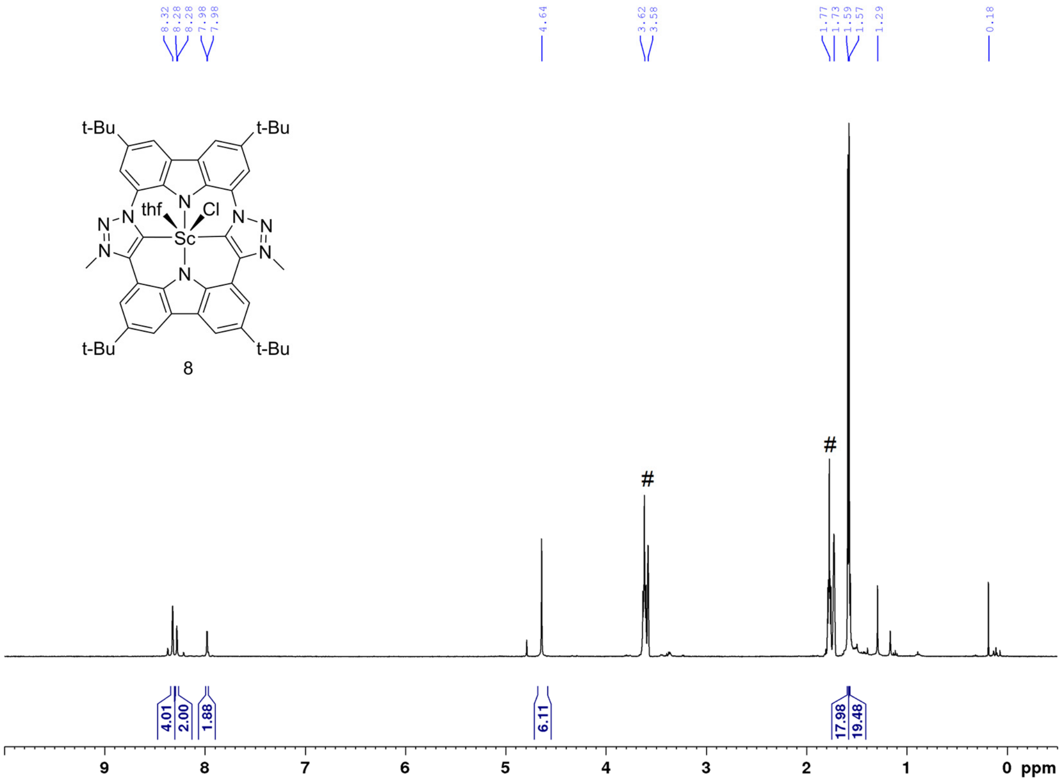


**Figure S20.** $^{1}$H NMR (THF-d$_8$ (#), 400 MHz) spectrum of complex **8**.

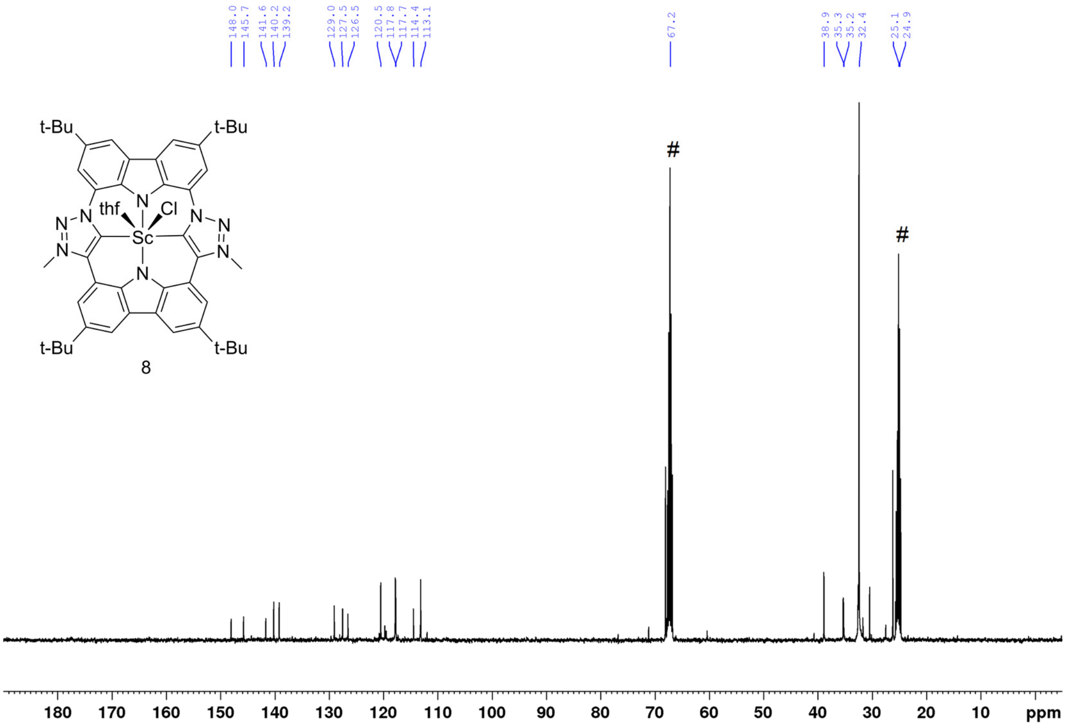


**Figure S21.** $^{13}C$ NMR (THF-$d_8$ (#), 100 MHz) spectrum of complex **8**.

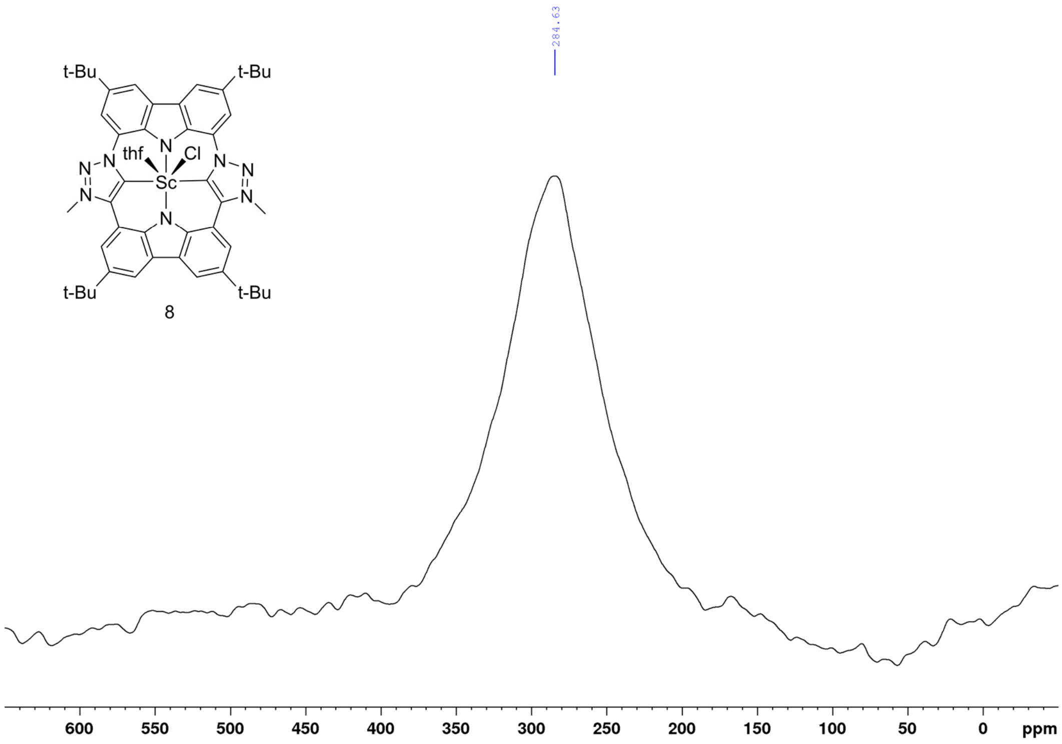


**Figure S22.** $^{45}Sc$ NMR (THF-$d_8$, 121 MHz) spectrum of complex **8**.

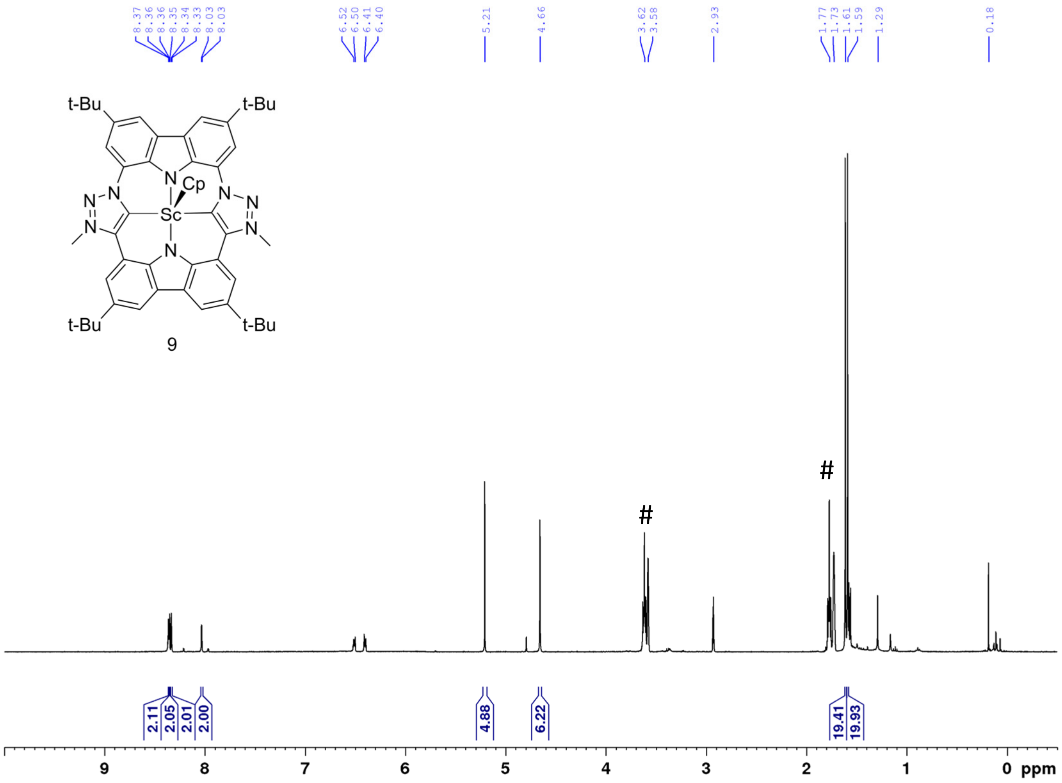


**Figure S23.** $^1$H NMR (THF-$d_8$ (#), 400 MHz) spectrum of complex **9**.

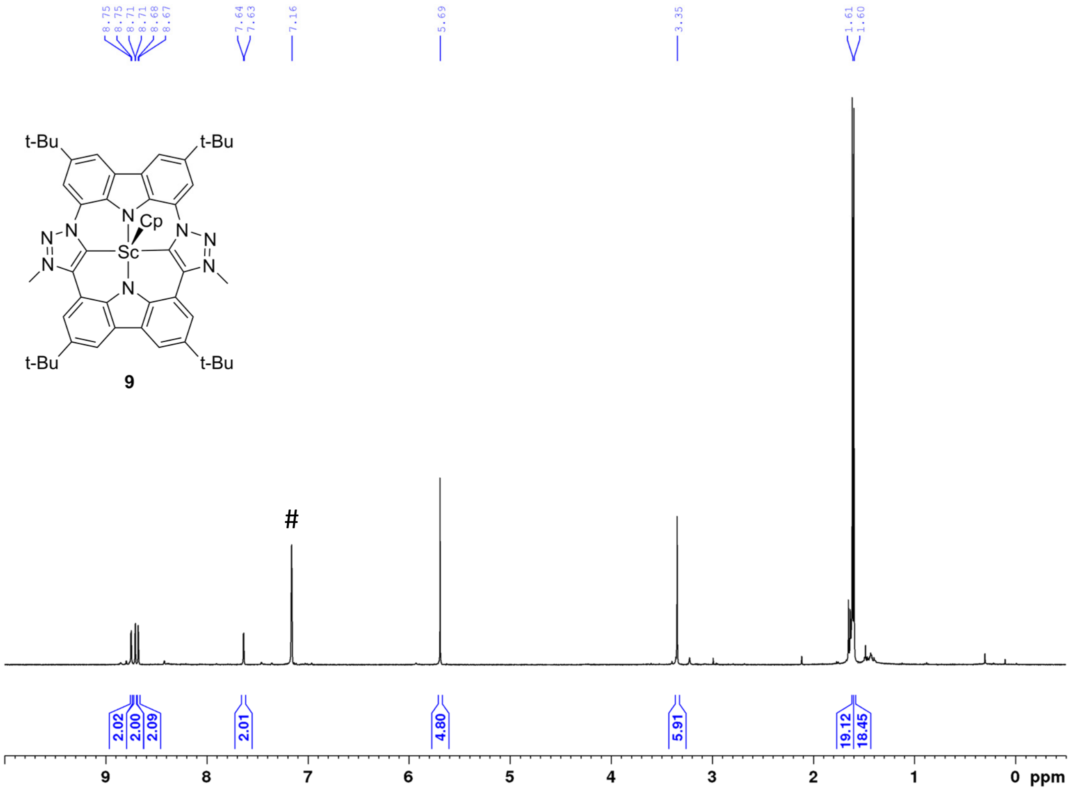


**Figure S24.** $^1$H NMR ($C_6D_6$ (#), 400 MHz) spectrum of complex **9**.

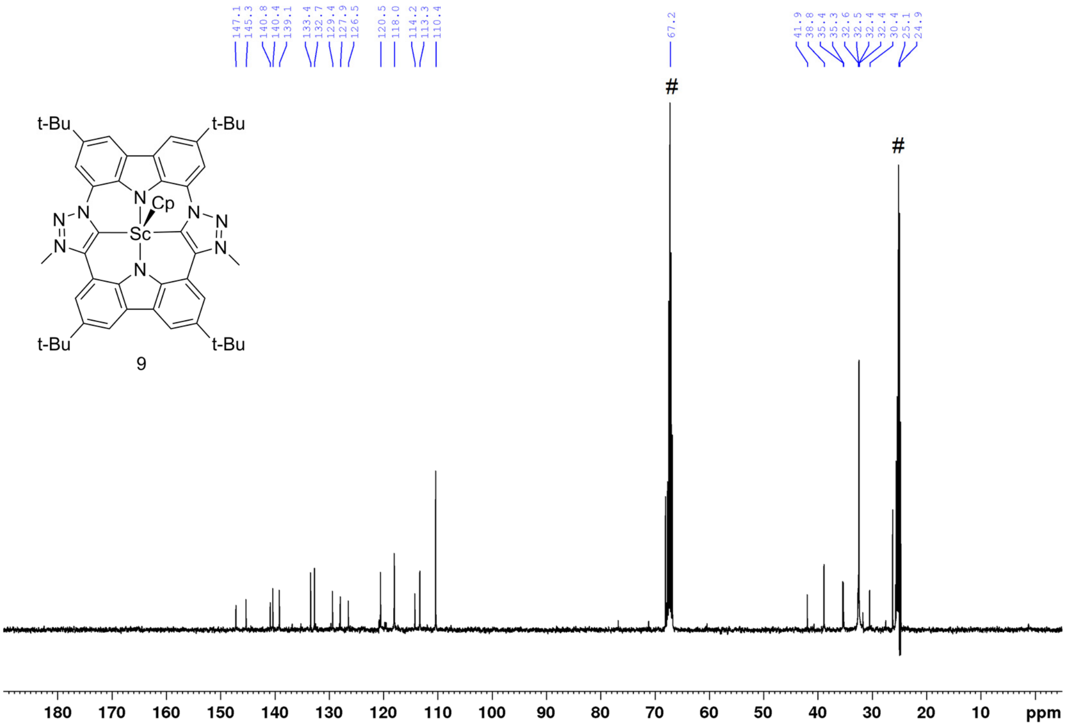


**Figure S25.** $^{13}C$ NMR (THF-$d_8$ (#), 100 MHz) spectrum of complex **9**.

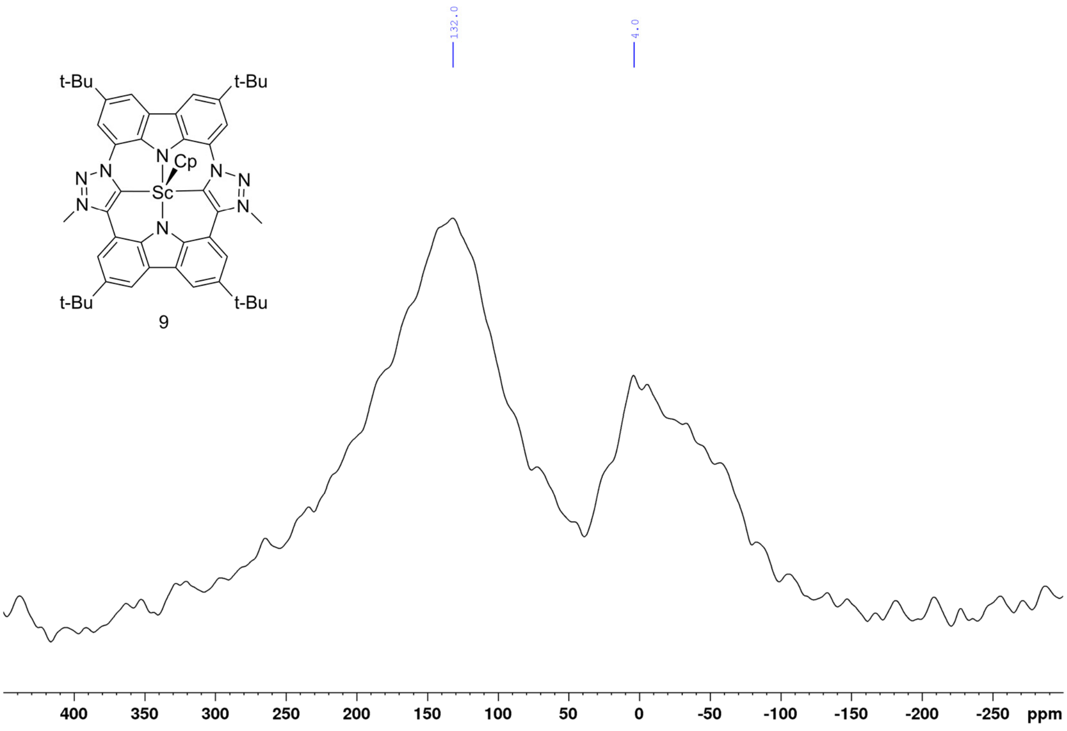


**Figure S26.** $^{45}Sc$ NMR (THF-$d_8$, 121 MHz) spectrum of complex **9**.

# 7. DFT Calculations

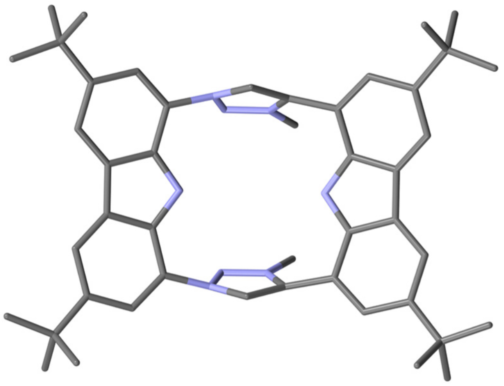

**Figure S27.** Geometry optimized structure of the "free" ligand **1** (DFT-D3BJ, BP86/def2-TZV).

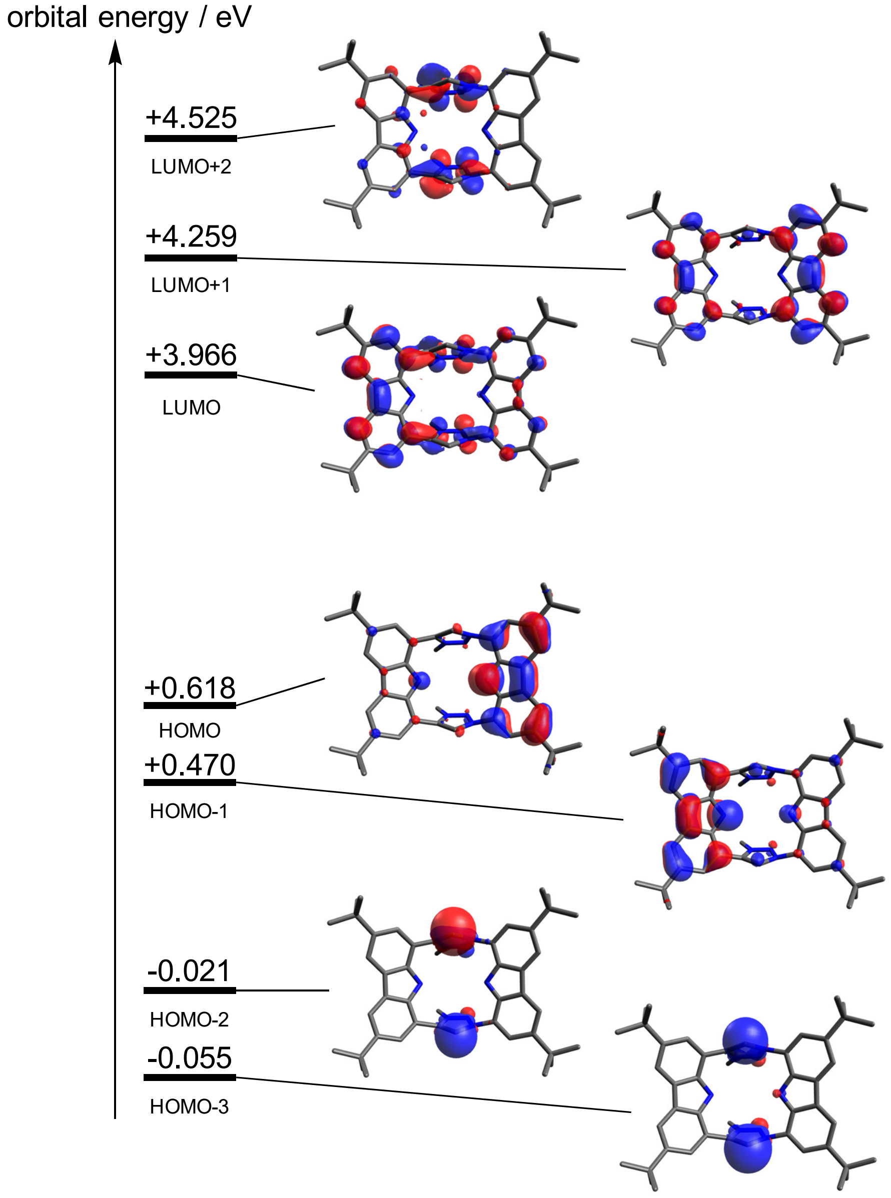


**Figure S28.** Schematic representation of the orbital energies of **1** at an iso value of 0.03 (DFT-D3BJ, BP86/def2-TZVP).

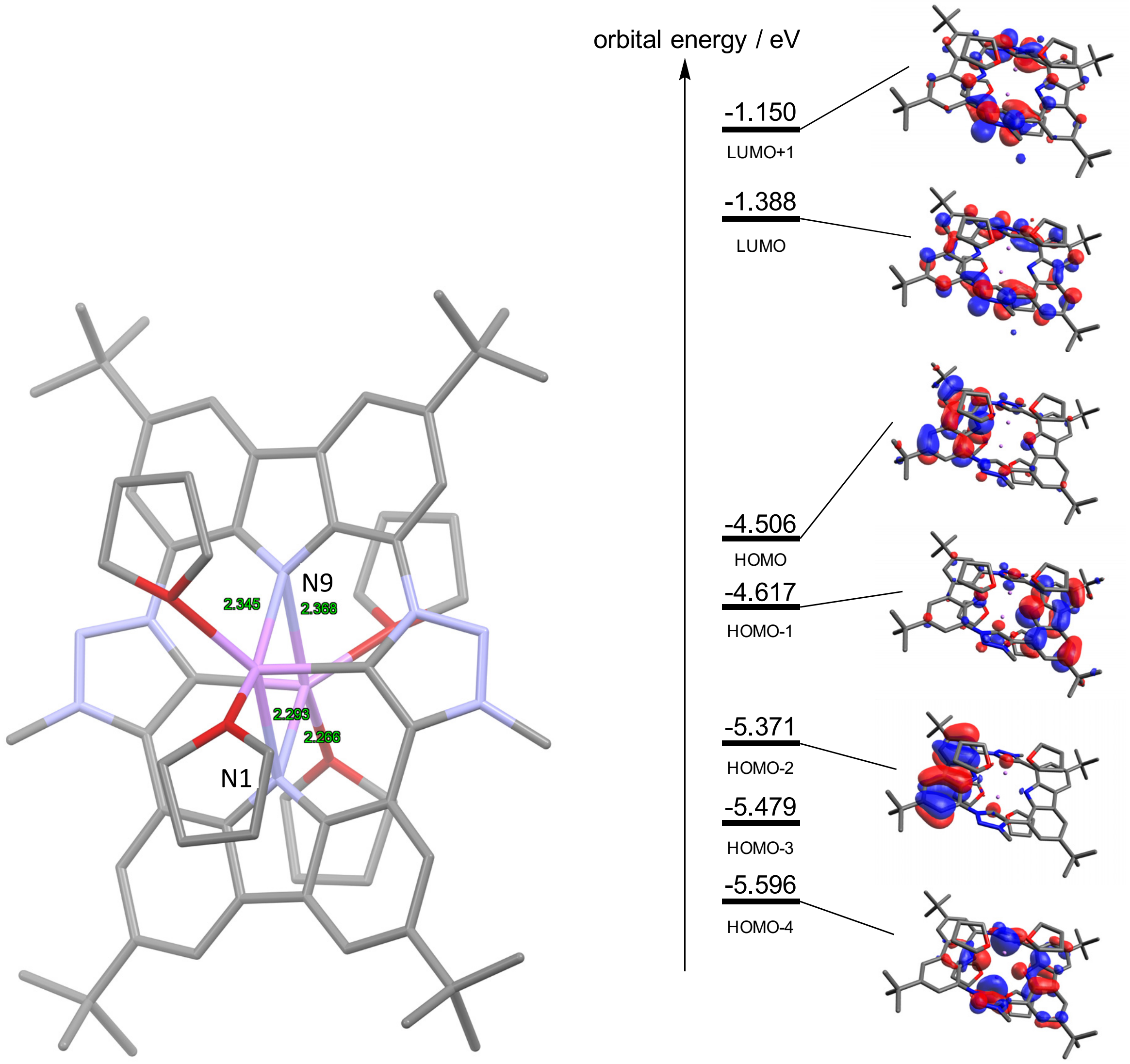


**Figure S29.** Left: Geometry optimized structure of complex **7** (DFT-D3BJ, BP86/def2-TZVP, CPCM (THF)) showing $\eta^3$-coordination of the CTP ligand to each $Li^+$ as well as two THF molecules to each $Li^+$. The Li-N bond lengths (green, in Å) are slightly shorter to N19 than to N9. Right: Schematic representation of the orbital energies at an iso value of 0.03.

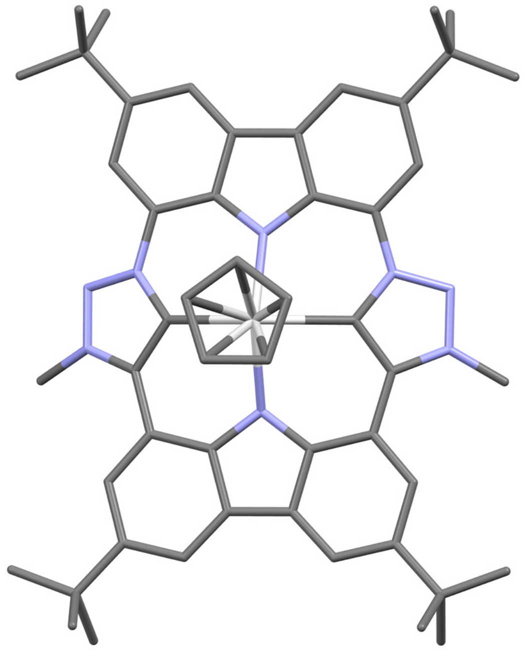

**Figure S30.** Geometry optimized structure of complex **9** (DFT-D3BJ, BP86/def2-TZV).

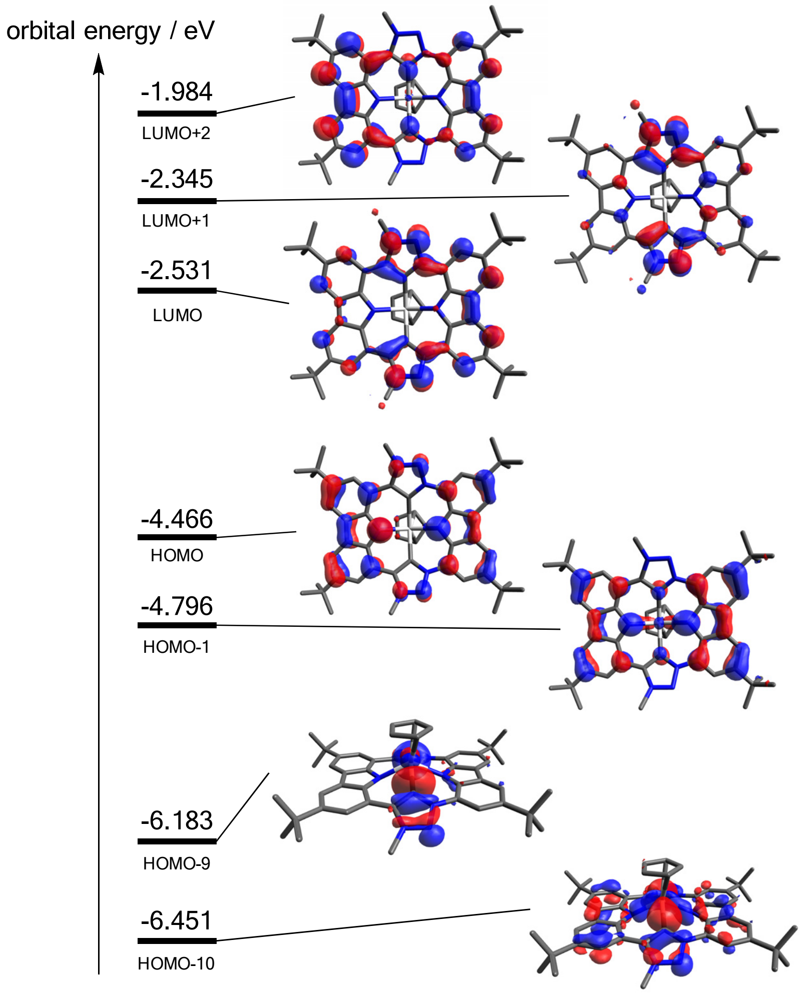


**Figure S31.** Schematic representation of the orbital energies of **9** at an iso value of 0.03 (DFT-D3BJ, BP86/def2-TZVP).

## Oxidization to the formally 18 e⁻ π-system

The oxidation of ligand **1** and its precursor **6** to an 18 πe⁻ macrocycle was investigated theoretically. The geometry optimization of the oxidized ligand **1$_{ox}$** did not lead to a minimum structure. However, the triazolium system **6$_{ox}$-2H$^+$**, which is similar to the oxidized pyrrole-carbazole porphyrinoid synthesized by Müllen and coworkers,[12] did converge to the structures **6$_{ox}$-2H$^+$-(S)** (singlet) and **6$_{ox}$-2H$^+$-(T)** (triplet) (Figure S33). The triplet state is lower in energy by 1.7 kcal/mol.

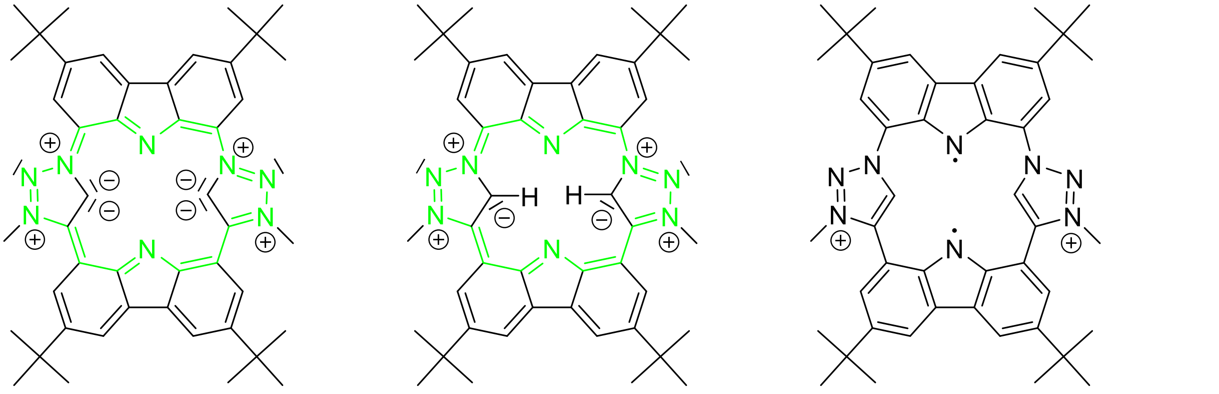

**1$_{ox}$** | **6$_{ox}$-2H$^+$-(S)** | **6$_{ox}$-2H$^+$-(T)**

**Figure S32.** Structure of the oxidation products **1$_{ox}$** and **6$_{ox}$-2H$^+$** (in singlet **(S)** and triplet **(T)** spin state) with a formal 18 e⁻ π-system.

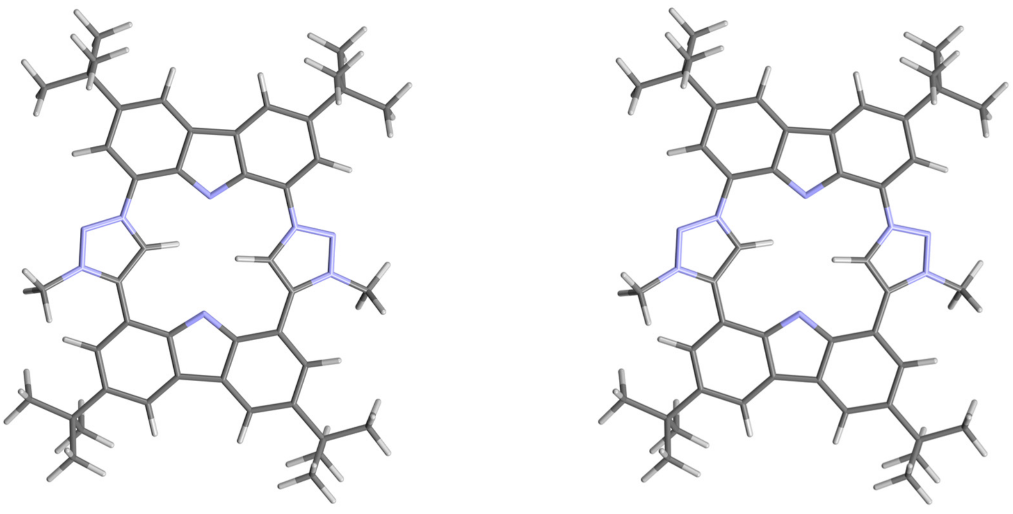

**Figure S33.** The geometry optimized structures of the oxidation product **6$_{ox}$-2H$^+$** (in singlet **(S)** and triplet **(T)** spin state) (DFT-D3BJ, BP86/def2-TZVP) show only slight differences in geometry. $E_{total}$ = -1394779.287 kcal/mol (**6$_{ox}$-2H$^+$-(S)**) and -1394777.606 kcal/mol (**6$_{ox}$-2H$^+$-(T)**); ΔE = 1.7 kcal/mol in favor of the triplet state.

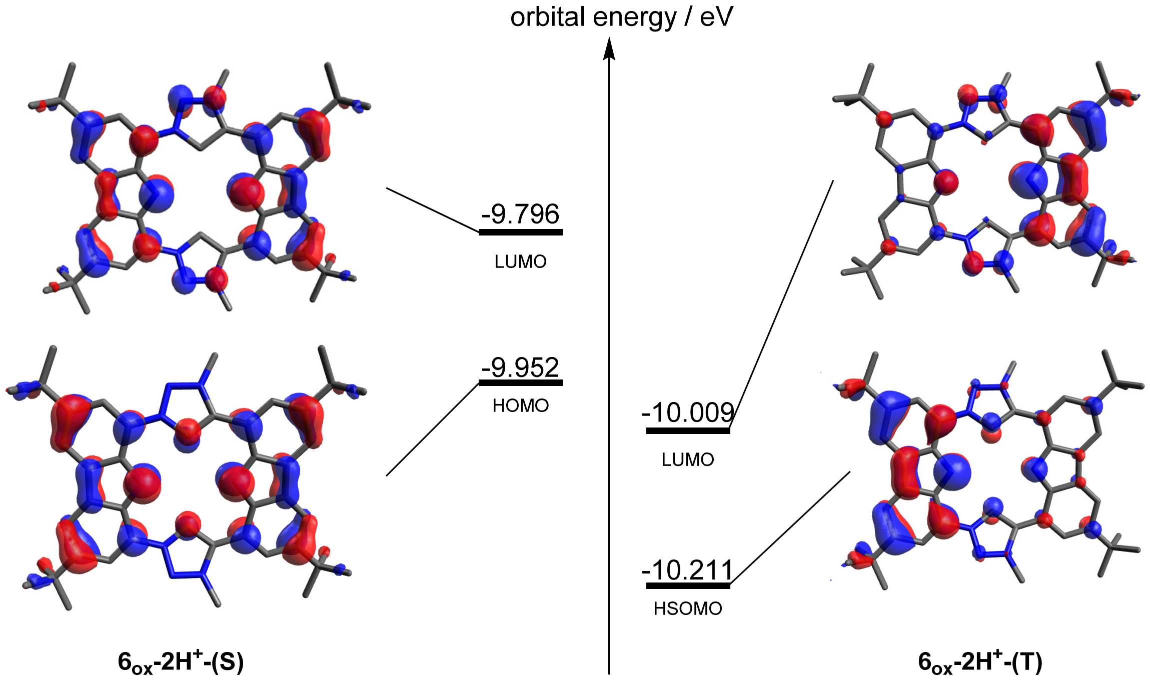


**Figure S34.** Schematic representation of the orbital energies of the geometry optimized structures of the oxidation product **$6_{ox}$-2H$^+$** (in singlet **(S)** and triplet **(T)** spin state) at an iso value of 0.03 (DFT-D3BJ, BP86/def2-TZVP).

# 8. References


[1] W. L. F. Armarego, C. L. L. Chai, Purification of laboratory chemicals, 6th ed.; Elsevier/BH: Oxford, 2009.

[2] Y. Liu, M. Nishiura, Y. Wang, Z. Hou, *J. Am. Chem. Soc.* **2006**, *128*, 5592–5593

[3] M. S. Bennington, H. L. C. Feltham, Z. J. Buxton, N. G. White, S. Brooker, *Dalton Trans.* **2017**, *46*, 4696–4710.

[4] G. M. Sheldrick, SADABS 2012/1; University of Göttingen, Göttingen, Germany, **2012**.

[5] a) G. M. Sheldrick, *Acta Cryst.* **2008**, *A64*, 112−122; b) G. M. Sheldrick, *Acta Cryst.* **2015**, *C71*, 3−8; c) C. B. Hübschle; G. M. Sheldrick; B. Dittrich, *J. Appl. Cryst.* **2011**, *44*, 1281−1284.

[6] a) F. Neese, *WIREs Comput. Mol. Sci.* **2018**, 8:e 1327. b) F. Neese, F. Wennmohs, U. Becker, C. Riplinger, *J. Chem. Phys.* **2020**, *152*, 224108-1.

[7] a) F. Weigend, *Phys. Chem. Chem. Phys.* **2002**, *4*, 4285–4291 b) F. Neese, F. Wennmohs, A. Hansen, U. Becker, *Chem. Phys.* **2009**, *356*, 98–109. b) S. Kossmann, F. Neese, *Chem. Phys. Lett.* **2009**, *481*, 240–243.

[8] a) A. D. Becke, *Physical Rev. A* **1988**, *38*, 3098–3100; b) J. P. Perdew, *Phys. Rev. B* **1986**, *33*, 8822–8824; c) C. Lee, W. Yang, R. G. Parr, *Phys. Rev. B* **1988**, *37*, 785–789. d) S. Grimme, S. Ehrlich, L. Goerigk, *J. Comput. Chem.* **2011**, *32*, 1456–1465; e) S. Grimme, J. Antony, S. Ehrlich, H. Krieg, *J. Chem. Phys.* **2010**, *132*, 154104-1–154104-19; f) F. Weigend, R. Ahlrichs, *Phys. Chem. Chem. Phys.* **2005**, *7*, 3297–3305.

[9] F. Neese, G. Olbrich, *Chem. Phys. Lett.* **2002**, *362*, *170*, 034107-1–034107-13.

[10] D. Zornik, R. M. Meudtner, T. El Malah, C. M. Thiele, S. Hecht, *Chem. Eur. J.* **2011**, *17*, 1473–1484.

[11] L. Arnold, *PhD thesis*, Johannes Gutenberg-Universität Mainz, Mainz, 2012.

[12] L. Arnold, M. Baumgarten, K. Müllen, *Chem. Commun.* **2012**, *48*, 9640–9642.
